# NanoSHP099‐Targeted SHP2 Inhibition Boosts Ly6C^low^ Monocytes/Macrophages Differentiation to Accelerate Thrombolysis

**DOI:** 10.1002/advs.202308166

**Published:** 2024-01-21

**Authors:** Kejing Ying, Wanghao Xin, Yiming Xu, Dandan Lv, Huiqi Zhu, Yeping Li, Wangting Xu, Chao Yan, Yiqing Li, Hongqiang Cheng, Enguo Chen, Guofeng Ma, Xue Zhang, Yuehai Ke

**Affiliations:** ^1^ Department of Pulmonary and Critical Care Medicine Regional Medical Center for National Institute of Respiratory Diseases Sir Run Run Shaw Hospital School of Medicine Zhejiang University Hangzhou 310016 China; ^2^ Department of Respiratory First Affiliated Hospital School of Medicine Zhejiang University Hangzhou China; ^3^ Department of Pathology and Pathophysiology Zhejiang University School of Medicine Hangzhou Zhejiang 310058 China; ^4^ Department of Pathology and Pathophysiology and Department of Respiratory Medicine at Sir Run Run Shaw Hospital Zhejiang University School of Medicine Hangzhou Zhejiang 310058 China

**Keywords:** collagenolysis, Liposomes, Ly6C^low^ monocytes/macrophages, SHP099, thrombolysis

## Abstract

Tumor‐associated thrombus (TAT) accounts for a high proportion of venous thromboembolism. Traditional thrombolysis and anticoagulation methods are not effective due to various complications and contraindications, which can easily lead to patients dying from TAT rather than the tumor itself. These clinical issues demonstrate the need to research diverse pathways for adjuvant thrombolysis in antitumor therapy. Previously, the phenotypic and functional transformation of monocytes/macrophages is widely reported to be involved in intratribal collagen regulation. This study finds that myeloid deficiency of the oncogene SHP2 sensitizes Ly6C^low^ monocyte/macrophage differentiation and can alleviate thrombus organization by increasing thrombolytic Matrix metalloproteinase (MMP) 2/9 activities. Moreover, pharmacologic inhibition by SHP099, examined in mouse lung metastatic tumor models, reduces tumor and thrombi burden in tumor metastatic lung tissues. Furthermore, SHP099 increases intrathrombus Ly6C^low^ monocyte/macrophage infiltration and exhibits thrombolytic function at high concentrations. To improve the thrombolytic effect of SHP099, NanoSHP099 is constructed to achieve the specific delivery of SHP099. NanoSHP099 is identified to be simultaneously enriched in tumor and thrombus foci, exerting dual tumor‐suppression and thrombolysis effects. NanoSHP099 presents a superior thrombus dissolution effect than that of the same dosage of SHP099 because of the higher Ly6C^low^ monocyte/macrophage proportion and MMP2/MMP9 collagenolytic activities in organized thrombi.

## Graphical abstract

After being injected into the tail vein of mice, NanoSHP099 specifically promotes the differentiation of Ly6C^low^ monocytes/macrophages, which highly express MMP2/MMP9, and increases their infiltration into thrombotic tissues, ultimately leading to the resolution of venous thrombi. Compared to the monomeric SHP099, NanoSHP099 is a more effective therapeutic agent for targeting and treating thrombotic lesions.

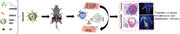

## 1 Introduction

Venous thromboembolism (VTE), including clinically diagnosed deep vein thrombosis (DVT) and pulmonary embolism (PE), is induced by various factors (malignant tumors, obesity, pregnancy, sedentary lifestyle, etc.), and is a major health problem with an annual incidence of approximately 1–2 cases per 1000 individuals.^[^ ^1^ ^]^ Among these VTE cases, tumor‐associated thrombus (TAT) accounts for nearly 20% of cases and has become the second leading cause of death in tumor patients.^[^ ^2^, ^3^ ^]^ Nonetheless, common thrombolytic drugs are limited by narrow therapeutic windows,^[^ ^4^, ^5^ ^]^ unsatisfactory effects, nonspecific bleeding,^[^ ^6^, ^7^ ^]^ and thrombocytopenia.^[^ ^8^ ^]^ Therefore, it is of great significance to develop new strategies that promote thrombolysis effectively and safely.

Because antithrombotic drugs cannot completely prevent thrombotic events, there are other thrombogenic mechanisms, such as inflammation, that can lead to treatment failure.^[^ ^9^, ^10^, ^11^ ^]^ Accumulating evidence indicates that thrombus resolution and collagenolysis are mediated by macrophage phenotypic transformation and their associated proteolytic enzymes, such as tissue‐type or urokinase‐type plasminogen activator (PLAT or PLAU) and matrix metalloproteinases (MMPs).^[^ ^12^, ^13^, ^14^, ^15^, ^16^ ^]^ The IL6/Stat3 signaling pathway,^[^ ^13^ ^]^ TNFα‐TNF/Rp55 axis,^[^ ^14^ ^]^ and IFNγ absence^[^ ^16^ ^]^ were revealed to induce macrophages expressing PLAU, MMP2, and MMP9 to accelerate thrombus dissolution. Additionally, augmentation of macrophage p53 activity using quinacrine‐promoted thrombolysis was found to be attributable to enhanced M2‐like phenotype polarization, which was associated with elevated MMP2 expression.^[^ ^17^ ^]^ These phenomena highlight the potential utility of macrophages in disrupting the synergy between inflammation and thrombosis.

Monocytes, the precursors of macrophages, comprised of inflammatory (classical) and patrolling (nonclassical) subsets, seem to play preemptive roles in the thrombus process. Inflammatory monocytes (in mice, Ly6C^high^CCR2^high^; in humans, CD14^high^CD16^low^) have been shown to promote inflammation, while patrolling monocytes (in mice, Ly6C^low^CX3CR1^high^; in humans, CD14^low^CD16^high^) are more regenerative, reputedly derived from Ly6C^high^ and then mature into macrophages, exerting pro‐healing and inflammation‐resolving activities.^[^ ^18^, ^19^ ^]^ Studies in a DVT mouse model showed that Ly6C^low^ monocytes/macrophages were essential for thrombolysis,^[^ ^20^ ^]^ and the recruitment of Ly6C^high^ monocytes contributed to thrombogenesis.^[^ ^21^, ^22^ ^]^ However, the exact contributions of different monocyte/macrophage subpopulations to thrombus progression are currently unclear.

The tyrosine phosphatase SHP2, encoded by the oncogene *PTPN11*,^[^ ^23^ ^]^ an early identified proto‐oncoprotein, has been extensively studied regarding its functions in supporting the malignant behaviors of tumor cells and inflammation immune regulation. There is a considerable amount of evidence suggesting that SHP2 loss can directly suppress the growth of tumor cells by restraining the RAS‐MAPK pathway or producing an antitumor immune microenvironment.^[^ ^23^, ^24^ ^]^ Additionally, as a highly specific inhibitor of SHP2, the anti‐oncogenicity and anti‐inflammatory effects of SHP099 have also been intensively identified. SHP099 administration not only cultivates antitumor immune responses of T cells^[^ ^25^, ^26^ ^]^ and tumor‐associated macrophages (TAMs)^[^ ^27^, ^28^ ^]^ but also protects against inflammatory diseases such as colitis, psoriasis, and neuroinflammation. Considering the influence of tumors on the thrombus process and inflammation intervention during thrombus evolution, it is reasonable to speculate that SHP2 inhibition triggering tumor suppression could ameliorate the thrombi burden. Nevertheless, the role of SHP2 in Ly6C^high^/Ly6C^low^ (another classification different from M1/M2) monocyte/macrophage subset transformation remains unknown. The efficacy of SHP099 in the context of thromboembolic inflammation still needs to be explored.

Here, we found that Ly6C^low^ monocytes/macrophages highly expressed metalloproteases, especially MMP2/MMP9, which conferred protection against thrombus organization. Myeloid‐restricted ablation of SHP2 in mice sensitized Ly6C^low^ monocyte/macrophage differentiation, increased intrathrombus Ly6C^low^ monocyte/macrophage infiltration and associated MMP2/MMP9 expression, thus lessening intrathrombus collagen deposition. Additionally, SHP2 blockade treated with the tumor inhibitor SHP099 in lung metastatic tumor mice was discovered not only to suppress tumor metastasis but also to reduce thrombi count and was further identified in a DVT mouse model to dose‐dependently decrease intrathrombus collagen, possessing a potential thrombolytic effect at its high dosage. Furthermore, we found that SHP099, which might be a new thrombolytic drug, administration could facilitate Ly6C^low^ monocyte/macrophage polarization by upregulating differentiation‐dependent C/EBPβ‐NR4A1 signaling. To significantly improve the thrombolytic effect of SHP099, we developed fibrin‐modified and SHP099‐loaded nanoliposomes (CREKA‐DiI‐Lipo@SHP099, called NanoSHP099). NanoSHP099 was confirmed to achieve its specific enrichment in tumor and thrombus foci, especially targeting intrathrombus monocytes/macrophages, and further verified in mouse thrombus and lung metastatic tumor models to potentiate better thrombolysis and tumor‐suppression effects in comparison to the same dosage of SHP099. All of these results suggest that NanoSHP099 possesses translational potential for favorable therapy for thrombotic events, particularly events in which thrombi are concurrent with tumors.

## 2 Results

### 2.1 Ly6C^low^ Monocytes/Macrophages with High MMP2/MMP9 Collagenolysis Activities are Important for Thrombolysis

**Figure 1:**
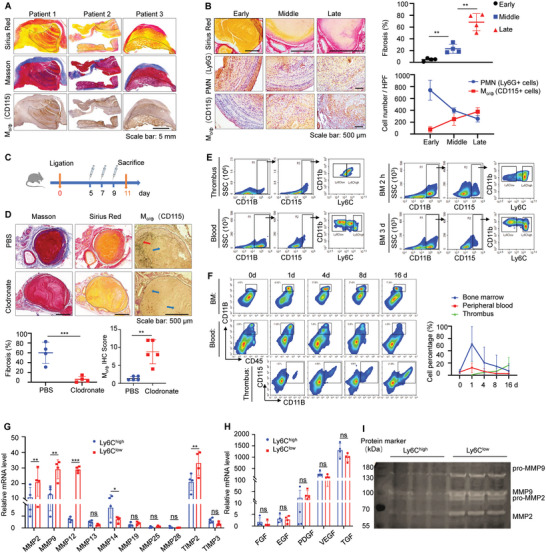
Ly6C^low^ monocytes/macrophages with high MMP2/MMP9 collagenolytic activities are important for thrombolysis A) Thrombus tissues exfoliated from VTE patients after pulmonary endarterectomy stained with Masson, Sirius Red, and CD115 immunohistochemistry to determine the locations of collagen and monocytes/macrophages. Scale bar: 5 mm. B) Left: Early, middle, and late stages of thrombus tissues acquired from DVT mouse models stained with Sirius Red, Ly6G, and CD115 immunohistochemistry to observe the dynamic changes of collagen and immune cells. Scale bar: 500 µm. Right: Intrathrombus fibrosis contents and cell numbers were measured by Image J. Data are shown as mean ± SD (n = 4). ^*^ *p* < 0.05, ^**^ *p* < 0.01 and ^***^ *p* < 0.001. C) Monocytes/macrophages systematic clearance with Chlorophosphate liposomes and the control groups with PBS liposomes by tail vein injection at the day 3 after DVT modeling and administering treatment every other day for total 4 times, then DVT mice were sacrificed at the day 11. D) Above: Thrombus tissues obtained from DVT mice after Chlorophosphate or PBS liposome treatments stained with Masson, Sirius Red, and CD115 immunohistochemistry to identify the differences of intrathrombus fibrosis contents. The red arrow shows a high expression of CD115, and the blue arrow shows a low expression of CD115. Scale bar: 500 µm. Below: Intrathrombus fibrosis contents were counted by Image J. Data are shown as mean ± SD (n = 4). ^*^ *p* < 0.05, ^**^ *p* < 0.01 and ^***^ *p* < 0.001. Immunohistochemical score was performed to test the monocytes/macrophage clearance efficiency in the thrombus tissues of DVT mice after Chlorophosphate liposome treatments, and PBS was used as the control group. E) Gating strategies to distinguish CD11B^+^CD115^+^Ly6C^high^ and CD11B^+^CD115^+^Ly6C^low^ cell clusters for qualitative analysis, and density plots of Ly6C monocytes/macrophages subsets in thrombi, peripheral blood, freshly isolated bone marrow, and bone marrow cells cultured in vitro for 3 days were presented by FCM. F) Left: Density plots of CD45^+^CD11B^+^ cell clusters in bone marrow, peripheral blood, and CD45^+^CD11B^+^CD115^+^ cell clusters in thrombi for qualitative analysis before inferior vena cava stenosis and days 1, 4, 8, 16 after stenosis dynamically detected by FCM. Right: The statistics data of percentages of myeloid cells (CD45^+^CD11B^+^) in bone marrow, peripheral blood, and monocytes/macrophages (CD45^+^CD11B^+^CD115^+^) in thrombi at different time points. G,H) Relative MMPs (MMP2, 9, 12, 13, 14, 19, 25, 28 and TIMP2, 3) (left) and GFs (FGF, EGF, PDGF, VEGF, and TGF) (right) mRNA levels in bone marrow separated Ly6C^high^ Ly6C^low^ monocytes/macrophages subsets were measured by qPCR. Data are shown as mean ± SD (n = 4). ^*^ *p* < 0.05, ^**^ *p* < 0.01 and ^***^ *p* < 0.001. I) Representative gel images of MMP2 and MMP9 activities in isolated Ly6C^high^ Ly6C^low^ monocytes/macrophages subsets as measured by gel zymography. (BCA protein quantification data can be found in Figure S1E, Supporting Information).

Thrombus organization characterized by intrathrombus collagen deposition is the key physiopathology resulting in thrombus insolubility.^[^ ^29^, ^30^, ^31^, ^32^ ^]^ Thrombus tissues endarterectomies from VTE patients were stained with Masson's trichrome and Sirius Red stains and subjected to CD115 immunohistochemistry. These refractory thrombi presented various degrees of organization, and the collagen deposition areas overlapped with the monocyte/macrophage distribution areas (**Figure** **1**A). To confirm the involvement of monocytes/macrophages in the thrombus organization process, different stages of thrombus tissues from the DVT mouse model were evaluated, and monocyte/macrophages accumulated along with collagen increase and gradually superseded neutrophils to become the dominant immune cells infiltrated in the late stage (Figure 1B). When total monocytes/macrophages were systematically removed from mice with thrombi by applying chlorophosphate liposomes as shown in Figure 1C (the clearance efficiency was confirmed by flow cytometry (FCM) in Figure S1A, Supporting Information). It can be seen that chlorophosphate liposome has a good monocyte/macrophage clearance efficiency, and the absence of monocytes/macrophages reduces the deposition of collagen in thrombotic tissue, making the structure of the thrombus more unconsolidated (Figure 1D). This phenomenon prompted us to reconsider the complex functions of different monocyte/macrophage subsets during thrombus evolution.

According to the Ly6C levels on the cell surface, monocytes/macrophages derived from the peripheral blood of mice with thrombi, thrombus, and freshly isolated or 3 days in vitro cultured bone marrow (BM) could all be clearly identified in the Ly6C highly expressed subgroup (Ly6C^high^), low expressed subgroup (Ly6C^low^) and their transition subgroup by FCM measurement (Figure 1E). Additionally, continuous FCM detection during mouse thrombosis displayed temporary fluctuations in monocytes in the bone marrow and peripheral blood after thrombus formation, indicating the replenishing intrathrombus monocyte/macrophage accumulation (Figure 1F). These alterations indicated the homology of monocytes/macrophages in bone marrow, peripheral blood, and thrombi. Accordingly, Ly6C^high^ and Ly6C^low^ monocyte subsets sorted from bone marrow were used to represent intrathrombus monocytes/macrophages and further detected by RNA sequencing (RNA‐seq) to explore their collagen regulatory function. As shown in the cluster and change trend analysis (Figure S1B,C, Supporting Information), as well as the specific fragments per kilobase million (FPKM) value summary (Figure S1D, Supporting Information), the Ly6C^low^ subset expressed significantly higher levels of collagen degradation‐associated genes, mainly the MMPs family, including MMP2/MMP9/MMP12, than the Ly6C^high^ subset. Then, qPCR verification of these differentially expressed genes displayed similar results: the Ly6C^low^ monocyte subpopulation had distinctly higher MMP2, MMP9, and MMP12 mRNA levels than the Ly6C^high^ subpopulation, but the collagen synthesis‐associated gene (TGF, FGF, VEGF, and PDGF) mRNA levels between these two groups were comparable (Figure 1G,H). In addition, the enzymatic activities of MMP2 and MMP9 in different subsets of monocyte lysates assessed by gel zymography further manifested the higher collagenolytic activities of Ly6C^low^ monocytes/macrophages (Figure 1I; Figure S1E, Supporting Information).

The above data suggested that total monocyte/macrophage infiltration promoted thrombus organization during thrombus progression; however, the Ly6C^low^ monocyte/macrophage subset with high MMP2/MMP9 collagenolysis activities facilitated later collagen degradation, contributing to subsequent thrombus resolution.

### 2.2 Myeloid‐Restricted SHP2 Loss Sensitized Ly6C^low^ Monocyte/Macrophage Differentiation to Alleviate Thrombus Organization

**Figure 2:**
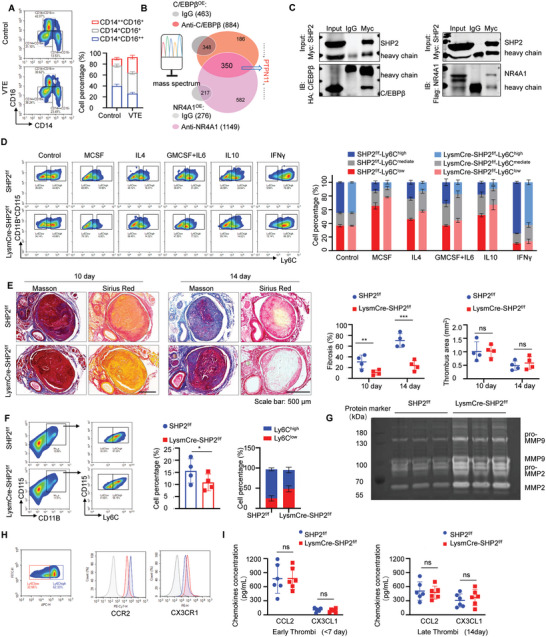
Myeloid‐restricted SHP2 loss sensitized Ly6C^low^ monocyte/macrophage differentiation to alleviate thrombus organization A) Left: Representative density plots of monocyte subpopulations derived from peripheral blood of VTE patients and healthy controls analyzed by FCM. Right: Stacking histogram of frequencies of CD14^++^CD16^+^, CD14^+^CD16^+^, and CD14^+^CD16^++^ monocytes subpopulations with some CD14^−^CD16^−^ cells not labeled, totaling 100%. B) Venn diagram analysis of overlapping genes among proteins that interact with NR4A1 and C/EBPβ. C) Myc‐tagged SHP2 and HA‐tagged C/EBPβ, or Myc‐tagged SHP2 and Flag‐tagged NR4A1 were respectively overexpressed in HEK293T cells. Co‐immunoprecipitation of SHP2 with anti‐SHP2 antibody to assess the presence of C/EBPβ or NR4A1. D) Left: Density plots showing the varies in proportions of CD11B^+^CD115^+^Ly6C^high^, CD11B^+^CD115^+^Ly6C^low^ monocytes/macrophages subsets in bone marrow suspension respectively derived from SHP2^f/f^ and LysmCre‐SHP2^f/f^ mice, after in vitro culture for 3 days with vehicle, MCSF (20 ng mL^−1^), IL4 (20 ng mL^−1^), GMCSF (10 ng mL^−1^) ^+^IL6 (10 ng mL^−1^), IL10 (20 ng mL^−1^), or IFNγ (20 ng mL^−1^) treatments. Right: Stacking histogram of proportions of Ly6C^high^ and Ly6C^low^ monocytes/macrophages in SHP2^f/f^ and LysmCre‐SHP2^f/f^ mice bone marrow suspension with different treatments. E) Left: Representative Masson and Sirius Red staining images of thrombus tissues obtained from SHP2^f/f^, LysmCre‐SHP2^f/f^ DVT mouse models at days 10, and 14 after inferior vena cava stenosis. Right: Intrathrombus fibrosis contents and thrombus areas were counted by Image J. Data are shown as mean ± SD (n = 4). ^*^ *p* < 0.05, ^**^ *p* < 0.01 and ^***^ *p* < 0.001. F) Left: Gating strategies to identify the total monocytes/macrophages (CD11B^+^CD115^+^) and Ly6C monocytes/macrophages subsets (CD11B^+^CD115^+^Ly6C^high^, CD11B^+^CD115^+^Ly6C^low^) in late thrombus tissues respectively from SHP2f/f and LysmCre‐SHP2f/f DVT mice. Right: The statistics data of frequencies of total intrathrombus monocytes/macrophages. Stacking histogram of proportions of intrathrombus Ly6C^high^ and Ly6C^low^monocytes/macrophages subsets. Data are shown as mean ± SD (n = 4). ^*^ *p* < 0.05, ^**^ *p* < 0.01 and ^***^ *p* < 0.001. G) Representative gel images of MMP2 and MMP9 activities in thrombus lysates respectively acquired from SHP2^f/f^ and LysmCre‐SHP2^f/f^ DVT mice as measured by gel zymography. (BCA protein quantification data can be found in Figure S1E, Supporting Information) H) Left: The density plots of Ly6c^low^ and Ly6c^high^ cells in the bone marrow macrophages of WT mice. Right: Showing the difference in expression levels of CCR2 and CX3CR1 between two groups of cells I) Serum levels of CCL2 and Cx3CL1 in SHP2f/f and LysmCre‐SHP2f/f DVT mice. Left: DVT mouse models less than 7 days. Right: DVT mouse models for 14 days. The serum levels of CCL2 and Cx3CL1 SHP2f/f and LysmCre‐SHP2f/f DVT mice were detected by ELISA assay. Data are shown as mean ± SD (n = 6). ^*^ *p* < 0.05, ^**^ *p* < 0.01 and ^***^ *p* < 0.001.

To study the possible role of Ly6C^high^/Ly6C^low^ monocyte/macrophage differentiation in thrombus progression, peripheral blood monocytes were isolated from VTE patients and healthy volunteers. The results showed higher CD14^++^CD16^+^ (equivalent to mouse Ly6C^high^) and lower CD14^+^CD16^++^ (equivalent to mouse Ly6C^low^) subgroup percentages analyzed by FCM (**Figure** **2**A) in VTE patient peripheral blood monocytes than in healthy controls. As has been widely reported, NR4A1 and C/EBPβ are two of the key regulators involved in Ly6C^low^ monocyte/macrophage differentiation,^[^ ^33^, ^34^, ^35^, ^36^, ^37^ ^]^ and the deficiency of either regulator stunted Ly6C^low^ monocyte/macrophage development. Actually, the relatively higher levels of NR4A1 and C/EBPβ in Ly6C^low^ monocytes/macrophages compared to those in the Ly6C^high^ subset were also verified by RNA‐seq (Figure S2A,B, Supporting Information). Subsequently, a proteomics assay was applied here for qualitative and quantitative analysis of the proteins concurrently interacting with NR4A1 and C/EBPβ. As the spectra showed, the gene encoding *PTPN11* for SHP2 was identified at the leading position among the 350 kinds of proteins concurrently binding with C/EBPβ and NR4A1 (Figure 2B) (the top 30 kinds of proteins synchronously combined with NR4A1 and C/EBPβ are listed in Figure S2C, Supporting Information).

In addition, the reverse CoIP assays (Figure 2C) further confirmed the interactions of C/EBPβ and NR4A1 with SHP2. It has been widely reported that the tyrosine phosphatase SHP2 participates in the regulation of platelet plug formation and stability. However, the function of SHP2 in other immune cells involved in thrombus evolution remains to be clarified. The above findings further identified the potential participation of SHP2 in Ly6C monocyte/macrophage subset differentiation.

Later, we introduced myeloid‐restricted SHP2 knockout mice (LysmCre‐SHP2^f/f^) and littermate control mice (SHP2^f/f^); the total amount and their respective proportions of Ly6C^high^/Ly6C^mediate^/Ly6C^low^ monocyte/macrophage subsets in bone marrow and peripheral blood were examined by FCM, presenting no difference between LysmCre‐SHP2^f/f^ and SHP2^f/f^ mice (Figure S2D,E, Supporting Information). However, the in vitro coculture of isolated bone marrow monocytes with various cytokine milieu showed that monocytes/macrophages with SHP2 deficiency tended to be more skewed toward Ly6C^low^ subgroup polarization (Figure 2D). Then, LysmCre‐SHP2^f/f^ and SHP2^f/f^ mice were subjected to DVT model induction, as was monitored in advance. SHP2 deficiency in monocytes/macrophages had no influence on the size of the early thrombus formation (Figure S2F, Supporting Information); nevertheless, intrathrombus collagen suppression was monitored in the developing thrombus at 10 and 14 days in LysmCre‐SHP2^f/f^ mice in comparison to SHP2^f/f^ mice (Figure 2E). Moreover, the total monocyte/macrophage decrease and Ly6C^low^ monocyte/macrophage subset proportion increase in LysmCre‐SHP2^f/f^ mouse thrombi, in contrast to those in SHP2^f/f^ mice, were measured by FCM (Figure 2F). Meanwhile, the collagenolytic activities of thrombus tissue lysates examined by gel zymography assays also revealed higher MMP2 and MMP9 enzyme activities in LysmCre‐SHP2^f/f^ mice than in SHP2^f/f^ mice (Figure 2G; Figure S1E, Supporting Information).

Notably, discrepancies of Ly6C^high^ monocyte/macrophages highly expressing CCR2, while the Ly6C^low^ subset highly expressing CX3CR1 (Figure 2H), lead to the former mainly responding to CCL2 recruitment while the latter responds to CX3CL1. By detecting CCL2 and CX3CL1 concentrations in thrombi at different stages by ELISA, it could be found that SHP2 deletion did not affect the intrathrombus abundances of CCL2 and CX3CL1, and CCL2 was much higher than CX3CL1 in both early and late thrombi (Figure 2I). These results excluded the interference of recruitment differences on intrathrombus Ly6C^high^/Ly6C^low^ monocyte/macrophage amounts; in addition, the total monocyte/macrophage declines in LysmCre‐SHP2^f/f^ mouse thrombi were attributed to their differentiation sensitization for Ly6C^low^ subsets with CCR2 expression decline, causing the weakening of main migration.

Collectively, the above data indicated that myeloid SHP2 loss sensitized Ly6C^low^ monocyte/macrophage polarization in thrombus tissues, thereby enhancing MMP2 and MMP9 activities to degrade intrathrombus collagen, which might be a promising therapeutic target in thromboembolic disease.

### 2.3 Administration of the Tumor Inhibitor SHP099 Promoted Thrombolysis in a Dose‐Dependent Manner

**Figure 3:**
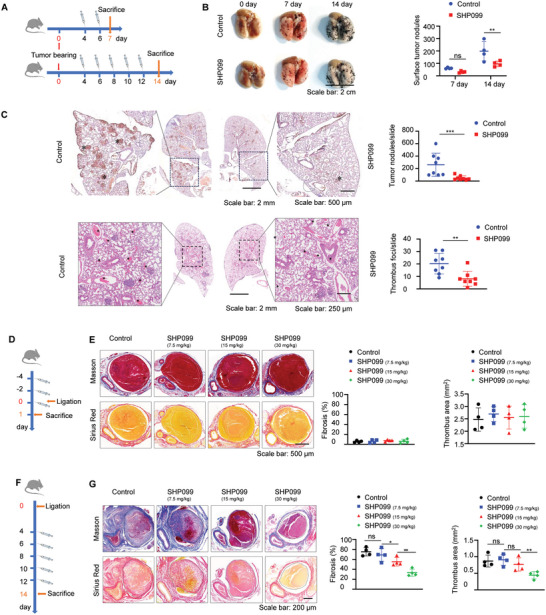
Administration of the tumor inhibitor SHP099 promoted thrombolysis in a dose‐dependent manner A) Lung metastatic tumor mouse models were established by tail intravenous injection of B16 melanoma cells, and treated with vehicle, SHP099 (15 mg kg^−1^) every other day from day 2 for total 7 or 14 days. Mice were sacrificed at day 7 and 14, respectively. B) Left: Representative images of lungs acquired from lung metastatic tumor mouse models sacrificed at day 7 and 14, respectively treated with vehicle or SHP099 (15 mg kg^−1^). Right: Visual counting of tumor nodules on the surface of mice lung tissue. Data are shown as mean ± SD (n = 4). ^*^ *p* < 0.05, ^**^ *p* < 0.01 and ^***^ *p* < 0.001. C) Above: Representative Ki67 immunohistochemistry images of whole lung tissues from lung metastatic tumor mouse models at day 7, respectively treated with vehicle or SHP099 (15 mg kg^−1^). The amplified images of outlined areas were further shown, and the lung metastatic tumor was marked with * (Only indicate individual items), Scale bar: 500 µm. Tumor foci per lung tissue section were counted by randomly selecting microscopic fields in Image J. Data are shown as mean ± SD (n = 4). ^*^ *p* < 0.05, ^**^ *p* < 0.01 and ^***^ *p* < 0.001. Below: Representative HE staining images showing thrombus lesions in lung tissues. Scale bar: 2 mm. The amplified images of outlined areas were further shown, and the thrombus lesion was marked with *, Scale bar: 250 µm. D) DVT mouse models were established by making inferior vena cava stenosis with thin line constraints, and treated with vehicle, SHP099 (7.5/15/30 mg kg^−1^) every other day from 4 days before being modeled for a total of three times, and mice were sacrificed at day 1 after modeled. E) Left: Representative Masson and Sirius Red staining images of early thrombus tissues acquired from DVT mouse models after treated with different doses of SHP099. Right: Intrathrombus fibrosis contents and thrombus areas were counted by Image J. Data are shown as mean ± SD (n = 4). ^*^ *p* < 0.05, ^**^ *p* < 0.01 and ^***^ *p* < 0.001. F) DVT mouse models were established by making inferior vena cava stenosis with thin line constraints and treated with vehicle, SHP099 (7.5/15/30 mg kg^−1^) every other day from day 3 after modeled for a total of five times, and mice were sacrificed at day 14 after modeled. G) Left: Representative Masson and Sirius Red staining images of late thrombus tissues acquired from DVT mouse models after treated with different doses of SHP099. Right: Intrathrombus fibrosis contents and thrombus areas were counted by Image J. Data are shown as mean ± SD (n = 4). ^*^ *p* < 0.05, ^**^ *p* < 0.01 and ^***^ *p* < 0.001.

SHP099, as a specific inhibitor of SHP2, is widely known for its high efficacy against tumors.^[^ ^23^, ^24^, ^38^, ^39^ ^]^ Tumors are the major high‐risk factor for thrombosis, and tumor‐associated thrombosis (TAT) has gradually become a major risk factor for VTE in cancer patients. Therefore, we extended our studies on the therapeutic effect of SHP099 from antitumor to antithrombin. Here, lung metastatic melanoma mice were generated, and the usual effective dose (15 mg kg^−1^) of SHP099 was intravenously administered as scheduled (**Figure** **3**A). The whole lungs acquired at Day 7 and 14 showed that lung tissues treated with SHP099 presented fewer metastatic foci than the control, especially on Day 14 (Figure 3B). The full‐scale lung sections examined by Ki67 histological staining also showed that SHP099‐treated mouse lung tissues developed significantly fewer tumor nodules than control mouse lung tissues. More notably, HE staining of lung tissues showed an obvious pulmonary thromboembolism decrease after SHP099 intervention compared with that of control mice (Figure 3C). We boldly propose a hypothesis that the antithrombotic effect of SHP099 is produced through a combination of indirect effects on reducing tumor burden and direct dissolution of thrombotic tissue. Nevertheless, in view of the thrombogenicity of tumor cells and the antitumor cell effects of SHP099,^[^ ^2^, ^3^ ^]^ it is unclear whether the reduction in thrombi can be directly attributed to enhanced thrombolysis triggered by SHP099. Hence, a DVT mouse model was further used to determine the direct influence of SHP099 on thrombus progression.

After administration of SHP099 at gradient dosages (0, 7.5, 15, 30 mg kg^−1^) following the schedules in Figure 3D,F, no significant difference in the size of the early formed thrombi of mice was observed in comparison to that in control mice (Figure 3E), while in the end‐stage thrombi, clot volumes and intrathrombus collagen contents gradually decreased as the dose of SHP099 administered increased until their final dissolution was treated with SHP099 at 30 mg kg^−1^ (Figure 3G).

Overall, apart from the high tumor‐suppression efficacy of SHP099, our observations utilized its emerging function of potentiating thrombolysis at high concentrations, which suggested that SHP099 might be a new antithrombin drug.

### 2.4 SHP099 Accelerated Ly6C^low^ Monocyte/Macrophage Differentiation by Upregulating C/EBPβ‐NR4A1 Levels

**Figure 4:**
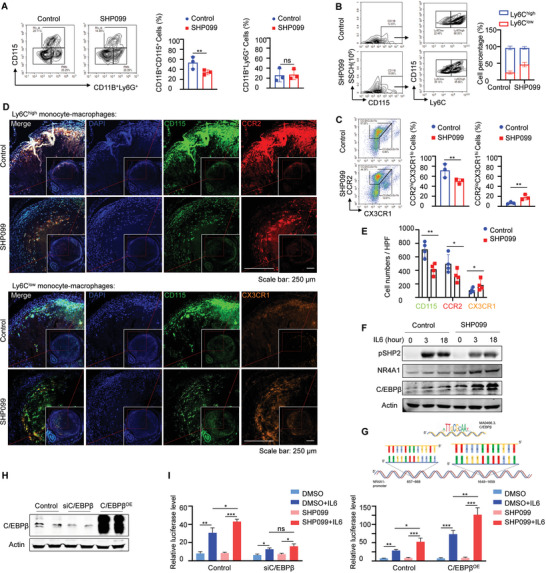
SHP099 accelerated Ly6C^low^ monocyte/macrophage differentiation by upregulating C/EBPβ‐NR4A1 levels A) Left: Contour plots of monocytes/macrophages (CD11B^+^CD115^+^) and neutrophils (CD11B^+^Ly6G^+^) in thrombi from SHP099 and control groups were gated to determine the immune cells infiltration analyzed by FCM. Right: The statistics data of percentages of intrathrombus monocytes/macrophages and neutrophils. Data are shown as mean ± SD (n = 3). ^*^ *p* < 0.05, ^**^ *p* < 0.01 and ^***^ *p* < 0.001. B) Left: Gating strategies to identify CD115^+^Ly6C^high^ and CD115^+^Ly6C^low^ monocytes/macrophages subsets in thrombi from SHP099 and control groups analyzed by FCM. Right: Stacking histogram of the proportions of intrathrombus Ly6C^high^ and Ly6C^low^ monocytes/macrophages subsets. Another gating strategy is to recognize intrathrombus Ly6C^high^ and Ly6C^low^ monocytes/macrophages subsets. C) Left: Another gating strategy to recognize intrathrombus Ly6C^high^ and Ly6C^low^ monocytes/macrophages subsets. CD115^+^CCR2^high^CX3CR1^low^ (namely Ly6C^high^ monocytes/macrophages) and CD115^+^CCR2^low^CX3CR1^high^ (namely Ly6C^low^ monocytes/macrophages) cell clusters in thrombi from SHP099 and control groups were gated in FCM. Right: The statistics data of percentages of intrathrombus CCR2^high^CX3CR1^low^ and CCR2^low^CX3CR1^high^ monocytes/macrophages. Data are shown as mean ± SD (n = 3). ^*^ *p* < 0.05, ^**^ *p* < 0.01 and ^***^ *p* < 0.001. D) Immunofluorescence staining for CD115 (green) and CCR2 (red), or CD115 (green) and CX3CR1(orange) in contiguous thrombus sections. Nuclei were counterstained with DAPI (blue). Scale bar: 250 µm. E) The statistics data of CD115, CCR2, or CX3CR1 positive cells were counted by randomly selected microscopic fields in Image J. Data are shown as mean ± SD (n = 4). ^*^ *p* < 0.05, ^**^ *p* < 0.01 and ^***^ *p* < 0.001. F) Western blotting showing pSHP2, NR4A1, and C/EBPβ levels in Raw264.7 stimulated by IL6 (50 ng mL^−1^) for 0, 3, and 18 h under the sustained treatment with vehicle or SHP099 (10 µm). Actin was used as a loading control. G) Two open chromatin regions upstream of the first exon of the NR4A1 gene locus were found harboring C/EBPβ binding motifs (NR4A1 promoter sequence and bold binding sites were listed). H) Western blotting identifying the knockdown and overexpression of C/EBPβ in HEK293T cells after transfected with C/EBPβ siRNA or pcDNA3.1‐C/EBPβ‐HA plasmids. Actin was used as a loading control. I) Luciferase activities of NR4A1 promoter were measured in C/EBPβ knockdown, overexpressed, as well as untreated HEK293T cells, stimulated with IL6 (50 ng mL^−1^) for 12 h with or without SHP099 sustained treatments. Luciferase activity is normalized to Renilla activity. Data are shown as mean ± SD (n = 4). ^*^ *p* < 0.05, ^**^ *p* < 0.01 and ^***^ *p* < 0.001. J) Two open chromatin regions upstream of the first exon of the NR4A1 locus carry the C/EBPβ Binding motif.

The thrombolytic effect of SHP099 prompted us to explore intrathrombus monocyte/macrophage changes influenced by SHP099 administration. By means of FCM detection, total monocyte/macrophage diminishment in SHP099 (15 mg kg^−1^)‐treated thrombi was examined when compared with that in the controls, whereas the percentages of intrathrombus neutrophils between these two groups were comparable (**Figure** **4**A). Based on the higher CCR2 expression on the Ly6C^high^ monocyte/macrophage surface and higher CX3CR1 expression on the Ly6C^low^ subset surface, two different FCM cell cluster gating strategies were applied to further monitor the influence of SHP099 on intrathrombus Ly6C^high^ and Ly6C^low^ monocyte/macrophage infiltration. As presented in Figure 4B,C, increased Ly6C^low^ and reduced Ly6C^high^ subset infiltration proportions after SHP099 administration were identified. Additionally, adjacent thrombus tissue sections were costained with CD115 and CCR2 or CD115 and CX3CR1 immunofluorescence for analysis of the absolute changes in the number of intrathrombus monocyte/macrophage subsets (Figure 4D). The number of low‐ratio Ly6C^low^ cells increased, even with the decrease in the number of high‐ratio Ly6C^high^ cells and overall monocytes/macrophages in SHP099‐treated thrombi, in contrast to controls (Figure 4E).

To further exploit Ly6C^low^ monocyte/macrophage differentiation after SHP099 treatment, Raw264.7 cells (mouse monocyte/macrophage line) under IL6 stimulation were detected by WB and used to determine that the C/EBPβ and NR4A1 proteins were upregulated when pSHP2 expression was suppressed by SHP099 (Figure 4F). Inspired by studies reporting that NR4A1 gene expression was strongly decreased in C/EBPβ‐deficient Ly6C^low^ monocytes and that there were binding sites on the NR4A1 promoter region that C/EBPβ could bind to and activate NR4A1 transcription,^[^ ^37^, ^40^, ^41^ ^]^ we reanalyzed the sequences of the mouse C/EBPβ and NR4A1 genes acquired from a public genome browser (https://jaspar.genereg.net/analysis). As simulated in Figure 4G, two open chromatin regions upstream of the first exon of the NR4A1 gene locus were found to harbor C/EBPβ binding motifs (the NR4A1 promoter sequence and bold binding sites are listed). Subsequently, C/EBPβ knockdown and overexpression constructs were generated in HEK293T cells by transfecting siRNA or overexpression plasmids, and the protein alterations were confirmed by WB (Figure 4H). Thereafter, dual‐luciferase reporter assays driven by the NR4A1 promoter response element were performed to show that SHP099‐induced transcriptional activation was suppressed upon C/EBPβ knockdown but markedly enhanced when C/EBPβ was overexpressed (Figure 4I). These discrepancies indicated that SHP099‐mediated NR4A1 protein elevation was mainly attributed to strengthening C/EBPβ‐induced NR4A1 transcription.

Overall, these data indicated that SHP099 administration promoted Ly6C^low^ monocyte‐macrophage differentiation, which might be ascribed to SHP2 activity inhibition to upregulate transcription Factor C/EBPβ and NR4A1 levels.

### 2.5 SHP099 Delivered by CREKA‐DiI‐Modified Liposomes Presented Great Characteristics and Biocompatibility

**Figure 5:**
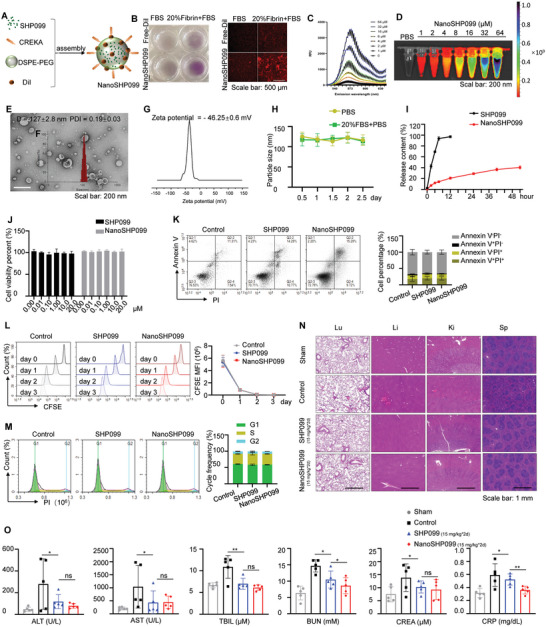
SHP099 delivered by CREKA‐DiI‐modified liposomes presented great characteristics and biocompatibility A) The schematic diagram of the compositions of NanoSHP099 liposomes. B) Representative realistic and fluorescent images of Free‐DiI, NanoSHP099 stuck to FBS or FBS+20% Fibrin. C) The fluorescence spectra of NanoSHP099 at various concentrations. D) Representative fluorescence image of NanoSHP099 at various concentrations irradiated by pulsed laser (549/565 nm). E) The TEM image of NanoSHP099. Scale bar: 200 nm. F) Representative dynamic light scattering of NanoSHP099 in ddH_2_O. G) Representative Zeta potential of NanoSHP099 in ddH_2_O. H) The liposome stability of NanoSHP099 in PBS and PBS+20% FBS over the time course of 2.5 days by dynamically recording the particle sizes. I) Kinetics of free SHP099 release from SHP099 and NanoSHP099 in 100% FBS. Data are shown as mean ± SD (n = 3). J) Cytotoxicity of different concentrations of SHP099 and NanoSHP099 on Raw264.7 after 24 h drug treatment. Data is shown as mean ± SD (n = 4). K) Effect of SHP099 and NanoSHP099 in equivalent concentration (10 µm) on apoptotic cell proportion in Raw264.7 detected by FCM using an Alexa Fluor 488 Annexin V/PI staining kit after 24 h drug treatment. L) Effect of SHP099 and NanoSHP099 in equivalent concentration (10 µm) on cell proliferation in CFSE dyed Raw264.7 over the time course of 3 days by dynamically detecting cell fluorescence intensities every day. M) Effect of SHP099 and NanoSHP099 in equivalent concentration (10 µm) on cell cycle distribution pattern in Raw264.7 detected by FCM after PI staining after 24 h drug treatment. N) Representative histological images of different organs acquired from DVT mouse models with tail vein injection of vehicle, SHP099 (15 mg kg^−1^) or NanoSHP099 (7.5, 15 mg kg^−1^). Scale bar: 1 mm. O) The statistics data of biochemical indexes including liver function (ALT, AST, TBIL), kidney (BUN, CREA), inflammation (CRP), fibrinolysis (FDP) in peripheral blood serum obtained from sham groups and DVT mouse models subjected to vehicle, SHP099 (15 mg kg^−1^) or NanoSHP099 (7.5, 15 mg kg^−1^) analyzed by ELISA. Data are shown as mean ± SD (n = 5). ^*^ *p* < 0.05, ^**^ *p* < 0.01 and ^***^ *p* < 0.001.

To improve thrombolytic efficiency, we prepared NanoSHP099 liposomes to concentrate SHP099 in thrombus lesions. As depicted in **Figure** **5**A, the hydrophobic small molecule compound SHP099 was encased in the lipophilic area of the phospholipid bilayer and further cloaked with polyethylene glycol 2000 (PEG2000) to prolong blood cycle time; moreover, NanoSHP099 was engineered with fibrin‐targeted small peptide CREKA and marked with DiI fluorescence to increase target and tracer abilities. The strong adhesion of NanoSHP099 onto fibrin‐coated slides reflected its high affinity for thrombi^[^ ^42^ ^]^ (Figure 5B). Additionally, NanoSHP099 generated peak fluorescence when irradiated with a 549/565 nm pulsed laser, and the fluorescence intensities gradually increased as the SHP099 concentration increased (Figure 5C,D).

As shown in the SEM image, NanoSHP099 exhibited characteristic orbicular structures, demonstrating the successful and complete self‐assembly of amphiphilic phospholipid molecules (Figure 5E). The average hydrodynamic diameter of NanoSHP099 was 127±2.8 nm at an average polymer dispersity index (PDI) of 0.19±0.03 when measured by a dynamic laser scattering system (DLS) (Figure 5F). The average surface zeta potential of NanoSHP099 was −46.25±0.6 mV in ddH_2_O (pH 7.4) (Figure 5G), and this shielding of negative charge could protect it from unspecific adhesion of plasma protein when cycled in blood. The particle sizes of NanoSHP099 remained relatively stable for two and a half days at 37°C in PBS or PBS containing 20% FBS (Figure 5H). The in vitro drug release patterns of NanoSHP099 and SHP099 showed that initially, rapid SHP099 burst release occurred on SHP099, while a small amount of SHP099 in NanoSHP099 was released within the same period (Figure 5I), and this sustained release property of NanoSHP099 was beneficial for maintaining the serum drug concentration.

Next, the biological safety of NanoSHP099 was evaluated in Raw264.7 cells cultured in vitro, and the results showed that there were no significant differences in cytotoxicity (Figure 5J), cell apoptosis (Figure 5K), cell proliferation (Figure 5L), or the cell cycle (Figure 5M) after treatment with SHP099, NanoSHP099 or the solvent control. Furthermore, HE staining of various tissues acquired from DVT model mice showed that there were some blood clots and structural injuries in control tissues but no obvious abnormalities in SHP099‐ or NanoSHP099‐administered tissues, confirming the in vivo safety of NanoSHP099 (Figure 5N). In addition, the serum biochemical indicators summarized in Figure 5O reflected deteriorated hepatic and renal function, as well as active inflammation after thrombosis modeling, but SHP099 or NanoSHP099 treatments could partly recover these parameters, especially the alleviation effects of NanoSHP099 on inflammation.

Overall, we successfully constructed NanoSHP099, and this new delivery method of SHP099 wrapped in CREKA‐DiI‐modified liposomes presented great characteristics and biocompatibilities in vivo and in vitro.

### 2.6 NanoSHP099 Was Targeted to Tumors and Thrombi and Was Mostly Engulfed by Intrathrombus Monocytes/Macrophages

**Figure 6:**
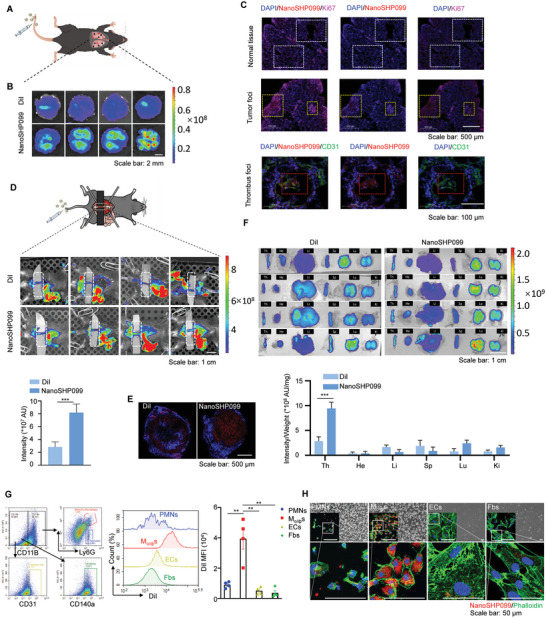
NanoSHP099 was targeted to tumors and thrombi and was mostly engulfed by intrathrombus monocytes/macrophages A) A lung metastasis model was established by injecting B16 tumor cells into the tail vein of mice. At day 7, NanoSHP099 was injected intravenously, and DiI was used as the control group. Then, the fluorescence intensity in DVT mice was measured one day after drugs administration. B) In vivo fluorescence images of the thoracic areas (Transverse section) in lung metastatic tumor mouse models treated with free‐DiI or NanoSHP099, respectively. C) Above: Immunofluorescence staining of Ki67 (pink) and NanoSHP099 (red) in continuous tumor tissue sections. Nuclei were counterstained with DAPI (blue). Scale bar: 250 µm. The white dotted boxes show normal lung tissue while the yellow dotted boxes show tumor foci. Below: Immunofluorescence staining of CD31 (green) and NanoSHP099 (red) in continuous thrombotic tissue sections. Nuclei were counterstained with DAPI (blue). Scale bar: 250 µm. The red dotted boxes show thrombus foci. D) Above: DiI‐labeled NanoSHP099 liposomes or free‐DiI solution (with the same DiI concentration of 10 µg mL^−1^) were intravenously injected into mouse models at day 7 after IVC stenosis. Middle: In vivo fluorescence images of the inferior vena cava thrombus (outlined by the white rectangles) in DVT mouse models treated with free‐DiI or NanoSHP099, respectively. Below: Quantification of fluorescence intensities of differently treated thrombus areas. Data is shown as mean ± SD (n = 4). Data are shown as mean ± SD (n = 4). ^*^ *p* < 0.05, ^**^p < 0.01 and ^***^ *p* < 0.001. E) Representative fluorescent images of thrombus sections respectively treated with free‐DiI or NanoSHP099. F) Above: Ex vivo fluorescence images of the DiI and NanoSHP099 in thrombus tissues and the major organs. Th, He, Li, Sp, Lu, and Ki represent thrombus, heart, liver, spleen, lung, and kidney, respectively. Below: Quantitative analysis of the mean fluorescence intensity per unit mass in each organ or tissue shown in the ex vivo images. Data is shown as mean ± SD (n = 4). ^*^ *p* < 0.05, ^**^p < 0.01 and ^***^ *p* < 0.001. G) Left: Gating strategies to identify monocytes/macrophages (CD11B^+^CD115^+^), neutrophils (CD11B^+^Ly6G^+^), endothelial cells (CD11B‐CD31^+^), and phorocytes (CD11B‐CD140a^+^) in NanoSHP099 treated thrombus tissues analyzed by FCM. Right: The overlaying peaks of intracellular DiI fluorescence intensities of different types of cells in thrombi. The statistics data of intracellular mean DiI fluorescence intensities. Data is shown as mean ± SD (n = 4). ^*^ *p* < 0.05, ^**^p < 0.01 and ^***^ *p* < 0.001. H) Left: Representative fluorescent images of intracytoplasmic NanoSHP099 particles in different kinds of cells after 1 h co‐culture with equal NanoSHP099. Cytoskeleton were stained with FITC‐phalloidine (green). Scale bar: 50 µm. Right: The statistics data of numbers of intracytoplasmic NanoSHP099 particles in each cell counted by randomly selecting microscopic fields in Image J. Data are shown as mean ± SD (n = 4). ^*^ *p* < 0.05, ^**^p < 0.01 and ^***^ *p* < 0.001.

Previously, liposomes were widely reported to accumulate in tumor lesions because of enhanced permeability and retention (EPR) effects, which prompted us to explore the distribution of NanoSHP099 in concurrent tumors with thrombi. As presented in **Figure** **6**A, NanoSHP099 and the same concentration of DiI dilution were intravenously injected into lung metastatic melanoma mice. The fluorescence intensities of lung tissues acquired from NanoSHP099‐treated mice were significantly higher than those of the DiI control (Figure 6B), and the further fluorescence measurement of various dissociated organs showed remarkable intensity in lung tissues (Figure S2G). Then, NanoSHP099‐administered tumor metastatic lung tissues were serially sectioned and stained with CD31 and Ki67 immunofluorescence to further determine the precise location of NanoSHP099. Compared with normal lung tissue, NanoSHP099 (red fluorescence) was not only clustered in tumor foci but also accumulated in thrombus clots (Figure 6C).

Therefore, DVT mouse models were further used to evaluate the ability of NanoSHP099 to target thrombi. The in vivo fluorescence images of NanoSHP099‐ or DiI‐treated thrombus mice showed that inferior vena cava (IVC) thrombi (marked with white dashed lines) exhibited strong fluorescence intensities after NanoSHP099 administration in comparison to the weak fluorescence in DiI‐treated groups, likely due to circulating DiI staining (Figure 6D). Additionally, the histological staining of separated thrombus sections also verified more red fluorescence in NanoSHP099‐treated thrombi than in DiI controls (Figure 6E). Furthermore, thrombi and other tissues were isolated from mouse models, and their fluorescence intensities were normalized by matching their respective weights. A similar result was identified: NanoSHP099‐treated thrombi had the highest relative fluorescence (absolute fluorescence/weight) (Figure 6F), which reflected the superior affinity of NanoSHP099 for thrombi.

Considering the existence of diverse cell types in thrombus tissues, it was essential to distinguish whether intrathrombus monocytes/macrophages were the cells most affected by NanoSHP099. Cell suspensions of NanoSHP099‐treated thrombus tissues were analyzed by FCM to measure the DiI fluorescence intensities of different types of intrathrombus cells (including neutrophils, monocyte/macrophages, endothelial cells, and fibroblasts). Monocytes/macrophages presented higher DiI fluorescence intensities than the other three types of cells (Figure 6G). Meanwhile, NanoSHP099 was cocultured in vitro with these four kinds of cells to again confirm the strongest phagocytosis of NanoSHP099 by monocytes/macrophages (Figure 6H).

In summary, our prepared NanoSHP099 could bi‐directionally target tumor and thrombus lesions; even in the presence of only thrombi, NanoSHP099 presented superior thrombus targeting and was mostly phagocytosed by intrathrombus monocytes/macrophages.

### 2.7 NanoSHP099 Further Boosted Ly6C^low^ Monocyte/Macrophage Differentiation to Exert Better Thrombolysis Effects

**Figure 7:**
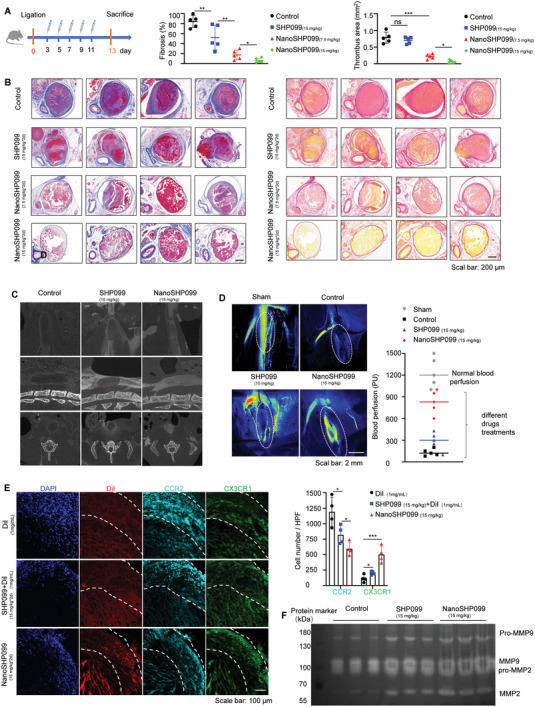
NanoSHP099 further boosted Ly6C^low^ monocyte/macrophage differentiation to exert better thrombolysis effects A) DVT mouse models were established by creating inferior vena cava stenosis and treated with vehicle, SHP099 (15 mg kg^−1^), NanoSHP099 (7.5/15 mg kg^−1^) every other day from day 3 after modeled for total five times, and mice were sacrificed at day 13 after modeled. B) Below: Masson and Sirius Red staining images of late thrombus tissues obtained from DVT mouse models subjected to various treatments. Scale bar: 500 µm. Above: Intrathrombus fibrosis contents and thrombus areas were quantitatively analyzed by Image J. Data is shown as mean ± SD (n = 5). ^*^ *p* < 0.05, ^**^p < 0.01 and ^***^ *p* < 0.001. C) CT images in multi‐dimensions of inferior vena cava of DVT mice after the various treatments of vehicle, SHP099 (15 mg kg^−1^), NanoSHP099 (15 mg kg^−1^). The inferior vena cava were outlined by the red circles.(First row: Median sagittal section; Second row: Coronal section; Third row: Transverse section) D) Left: Representative laser speckle flow imaging of inferior vena cava of DVT mice after the various treatments of vehicle, SHP099 (15 mg kg^−1^), NanoSHP099 (15 mg kg^−1^). The sham groups without modeled as the control. The inferior vena cava were outlined by the white circles. Right: The statistics data of mean blood perfusion of inferior vena cava in control groups and differently treated DVT mice. Data are shown as mean ± SD (n = 5). ^*^ *p* < 0.05, ^**^p < 0.01 and ^***^ *p* < 0.001. E) Immunofluorescence staining for CCR2 (green) (Ly6C^high^) and CX3CR1 (orange) (Ly6C^low^) in thrombi obtained from DVT mice subjected to various treatments. Nuclei were counterstained with DAPI (blue). The tissue within the two white dotted lines represents thrombi. Scale bar: 250 µm. F) Representative gel images of MMP2 and MMP9 activities in thrombus lysates respectively acquired from DVT mouse models respectively administrated with a vehicle, SHP099 (15 mg kg^−1^), and NanoSHP099 (15 mg kg^−1^) as measured by gel zymography.(BCA protein quantification data can be found in Figure S1E, Supporting Information).

Considering the dual‐target effects of NanoSHP099 on tumors and thrombi, its functions were explored in mice with lung metastatic tumors. CyTOF mass cytometry detection of metastatic lung tissue cell suspensions showed that NanoSHP099 treatment increased the infiltration of antitumor CD4^+^ and CD8^+^ T cells and regulated Ly6C^high^/Ly6C^low^ monocyte/macrophage changes, similar to SHP099 (Figure S3A, Supporting Information). Moreover, compared to SHP099, NanoSHP099 not only decreased lung nodules more significantly but also suppressed thrombi burden more markedly. To objectively compare the thrombolytic effects of NanoSHP099 and SHP099, these two drugs at the same dosage (7.5, 15 mg kg^−1^) were intravenously injected into DVT model mice as shown in **Figure** **7**A, and acquired thrombus tissues were adjacently stained with Masson and Sirius Red. The histological stains of thrombi showed that NanoSHP099 administration could achieve better collagenolysis and thrombolytic effects, leading to less collagen content and more evacuated structures, in comparison to SHP099 (Figure 7B).

Subsequently, the thrombolysis efficiencies of NanoSHP099 were further assessed by detecting hemodynamic changes. Micro‐CT scanning images in three dimensions showed that NanoSHP099 (15 mg kg^−1^) treatment allowed more contrast media to pass through the IVC vessels of the mouse models, whereas a small passage was observed in mice treated with the same concentration of SHP099 (Figure 7C). Meanwhile, mouse IVC blood perfusion measured by a laser speckle system also confirmed that little blood flow was recovered after SHP099 (15 mg kg^−1^) administration; nevertheless, obvious blood perfusion could be recovered after NanoSHP099 (15 mg kg^−1^) treatment (the normal blood flow standards were defined in sham mice) (Figure 7D). Meanwhile, NanoSHP099(15 mg kg^−1^) treatment reduced lung tumor levels and microthrombus foci in mouse models of lung metastases, and NanoSHP099 treatment was more effective than the same dose of SHP099 treatment (Figure S3B,C, Supporting Information)

Then, mice with thrombi were treated with DiI, SHP099+DiI, and NanoSHP099 at equal doses of SHP099 (15 mg kg^−1^) and DiI (1 mg kg^−1^). The immunofluorescence of thrombus tissues stained with CCR2 and CX3CR1 showed that NanoSHP099 targeted delivery generating drug accumulation in thrombi induced a more marked intrathrombus Ly6C^low^ monocyte/macrophage increase and Ly6C^high^ decrease than unspecific SHP099 administration (Figure 7E). Furthermore, a gel zymography assay of these thrombus tissue lysates revealed that MMP2 and MMP9 collagenolytic activities in NanoSHP099‐treated thrombi were also higher than those in SHP099‐treated thrombi due to the more intensive infiltration of intrathrombus Ly6C^low^ monocytes/macrophages (Figure 7F).

Conclusively, the above comparisons indicated the preferable thrombolytic efficiencies of NanoSHP099 over SHP099 due to its stronger ability to enhance intrathrombus Ly6C^low^ monocyte/macrophage accumulation and associated MMP2/MMP9 activities.

## 3 Discussion

Among the various risk factors for VTE, malignant tumors are the most common cause,^[^ ^2^, ^3^ ^]^ and TAT has become the second leading cause of death in tumor patients.^[^ ^3^ ^]^ This phenomenon not only indicates that effective thrombolysis could greatly improve the survival prognosis of tumor patients but also inspires us to develop a new therapeutic strategy from antitumor to combined antithrombin.

Recently, with the development of the “immune thrombosis” notion, many researchers have proposed new strategies to promote thrombolysis from the perspective of immune regulation. Macrophages, as highly heterogeneous immune cells, have been reported to relieve fibrosis by phenotypic remodeling to secrete collagenolytic enzymes.^[^ ^43^, ^44^, ^45^ ^]^ In our study, we focused on the functions of Ly6C^high^/Ly6C^low^ monocytes/macrophages, the precursors of macrophages, in thrombus progression. Previously, enhanced Ly6C^high^ monocyte recruitment in response to CCL2 was reported to promote thrombus formation,^[^ ^46^, ^47^ ^]^ and adoptive transfer of Ly6C^low^ monocytes/macrophages into monocyte/macrophage‐scavenging mice could reverse impaired thrombolysis; however, the exact mechanisms are still unknown.

We found significantly higher MMPs expression and activities, especially MMP2 and MMP9, by means of RNA‐seq, in Ly6C^low^ monocytes/macrophages, facilitating collagenolysis to ameliorate thrombus organization, emerging as a new target for thrombolysis. However, the levels of collagen synthesis‐associated genes, such as TGF, FGF, PDGF, and VEGF, between Ly6C^high^ and Ly6C^low^ monocytes/macrophages were nearly comparable, and these collagen synthesis‐associated genes, regardless of Ly6C^high^ or Ly6C^low^, were far higher than their collagen degradation‐associated genes. The above findings indicated that thrombus organization was the coincident effect of Ly6C^high^ and Ly6C^low^ monocytes/macrophages, but the high MMP2 and MMP9 levels in the Ly6C^low^ subset might be the key effectors for subsequent fibrinolysis. These two antagonistic effects of the Ly6C^low^ subset could explain why Ly6C^low^ macrophage differentiation abnormalities or survival limitations resulted in defective collagen healing in infarcted myocardium;^[^ ^48^ ^]^ however, adoptive transfer of Ly6C^low^ macrophages or their hepatic accumulation conferred liver fibrosis regression.^[^ ^49^ ^]^ These discrepant functions in collagen‐associated diseases were attributed to the stage at which Ly6C^low^ monocytes/macrophages come into play.

In our study, we introduced myeloid SHP2‐deficient mice for the wide involvement of the tyrosine phosphatase SHP2 in immune cell phenotypic remodeling and antitumor immunity. Previously, our research members found that macrophages lacking SHP2 were skewed toward M2 alternatively activated polarization and aggravated bleomycin‐induced fibrotic injury. However, another study designed a lipid nanoparticle (LNP) system loaded with amphipathic R848‐cholesterol (TLR7/8 agonist) and SHP099, which could repolarize M2 macrophages into M1 type to enhance the phagocytosis potential for macrophage immunotherapy in 4T1 tumor‐bearing mice.^[^ ^50^ ^]^ Subsequently, we found that SHP2 negatively regulated TGF‐β1–mediated extracellular matrix homeostasis and emphasized that SHP2‐deficient macrophages secrete more collagen‐elastase MMP12, degrading the extracellular matrix and resulting in spontaneous emphysema.^[^ ^51^ ^]^ These inconsistent conclusions may reflect the nonidentical functional requirements of SHP2 in different tissue structures and organs. Here, we focused on the role of SHP2 in Ly6C^high^/Ly6C^low^ monocyte/macrophage transformation in the context of pathological thrombus tissues. Our data demonstrated that SHP2 enzyme activity inhibition induced by SHP099 could elevate C/EBPβ‐NR4A1 expression to promote intrathrombus Ly6C^low^ subpopulation differentiation and their associated MMP2/MMP9 collagenolytic activities, thus relieving thrombus organization to facilitate thrombolysis. Distinct from the parallel classification of M1/M2 macrophage polarization, Ly6C^low^ monocytes/macrophages were reputedly differentiated from Ly6C^high^ monocytes and then matured into macrophages. Therefore, the classification of Ly6C^low^ monocytes/macrophages differed from M2 macrophages, and their functions also need to be clarified.

In subsequent studies, we adopted myeloid‐SHP2‐deficient mice and pharmaceutical SHP2 inhibition by SHP099 in DVT mouse models. Intrathrombus collagen content decreased, and the internal structure of thrombi was more evacuated in LysmCre‐SHP2^f/f^ mice. On the other hand, the thrombus organization degrees and clot volumes gradually decreased when treated with gradient concentrations of SHP099. This insufficient thrombolysis in myeloid‐SHP2‐deficient mice might be ascribed to inadequate gene knockdown efficiency compared to the reinforcing inhibition efficiency of SHP099 with increasing doses. Meanwhile, this phenomenon prompted us to explore a specific drug delivery method to concentrate SHP099 in intrathrombus monocytes/macrophages to improve thrombolytic efficiency.

By means of the peculiarity of monocyte/macrophage phagocytosis of exogenous particles,^[^ ^52^ ^]^ PEGylated liposomes with DiI fluorescence labeling were used as carriers of SHP099 and further modified with the fibrin‐targeting peptide CREKA, which has been widely reported in thrombus targeting studies.^[^ ^53^, ^54^, ^55^ ^]^ Additionally, on account of anionic phospholipids not only being the natural ligands of macrophage scavenger receptors but also reducing the nonspecific adsorption of negatively charged endothelial cells,^[^ ^56^, ^57^, ^58^ ^]^ NanoSHP099 was prepared as negatively charged liposomes. NanoSHP099 intensively accumulated in intrathrombus monocytes/macrophages to stimulate Ly6C^low^ subset differentiation and achieved more significant collagenolytic and thrombolytic effects than the same dosage of SHP099.

In our study, in addition to IVC stenosis‐induced DVT mouse models,^[^ ^59^, ^60^ ^]^ lung metastatic tumor mouse models were used to explore pulmonary tumor‐associated thrombi, considering the critical roles of tumors in the thrombosis process as well as the reported antitumor effects of SHP099. Regrettably, until now, there has been a lack of recognized and referable TAT animal models in experimental studies due to the uncertainty and probability of tumors triggering thrombus formation. Thereafter, the only unbiased and reliable method was to detect thrombus foci in whole lung tissues by serial slices. Interestingly, we found that SHP099 not only suppressed tumor metastasis but also decreased thrombi burden. Similarly, NanoSHP099 also possessed these dual therapeutic effects and was further enhanced for its dual‐targeting ability, not only to passively target tumors for the EPR effect of common liposomes but also to actively target thrombi due to CREKA adhering fibrin. Compared to SHP099, NanoSHP099 can achieve thrombolysis at a lower blood drug concentration, reducing the effects of systemic SHP2 inhibition on healthy organs and tissues.

Collectively, based on our findings that the antitumor drug SHP099 enhanced Ly6C^low^ monocyte/macrophage differentiation and associated fibrinolytic MMP2 and MMP9 expression to exert thrombolytic potential. We prepared NanoSHP099 liposomes to achieve specific SHP099 drug delivery, which exhibited better thrombus targeting abilities and therapeutic outcomes, thus providing a novel, safe, and promising approach for thrombosis treatment.

## 4 Experimental Section

### Experiment Reagents and procedures

Detailed experimental protocols are provided in the Supporting Information.

### Experimental Principles and Ethical Standards

C57BL/6 WT mice, and SHP2^f/f^, LysmCre‐SHP2^f/f^ transgenic mice used for animal studies were randomly assigned to different groups, and all samples were analyzed by experienced histopathologists who were blinded to the experimental conditions. For each experiment, the sample size reflected the number of independent biological replicates and was indicated in the figure legends.

All animal experimental procedures applications were approved by the Ethics Committee of Sir Run Run Shaw Hospital, Zhejiang University School of Medicine (approval number: SRRSH202102100).

All sample applications were approved by the Ethics Committee of Sir Run Run Shaw Hospital, Zhejiang University School of Medicine (approval number 2022‐0080).

### Statistics Analysis

Data are presented as mean ± standard deviation (SD). Statistical analysis was conducted using a One‐way ANOVA (for multigroup comparison) or Two‐tailed Student's *t*‐test (for two groups) by GraphPad Prism software version 7.0. Significance was reported as ^*^ when *p* < 0.05, ^**^ when *p* < 0.01, and ^***^ when *p* < 0.001. For nanoliposome characterization and in vitro or in vivo studies, all experiments were repeated independently at least three times with similar results.

## Conflict of Interest

The authors declare no conflict of interest.

## Author Contributions

K.Y., W.X., and Y.X. contributed equally to this work. K.Y., Y.K., and X.Z. were involved in the study design and Y.K. provided transgenic SHP2^f/f^ and LysmCre‐SHP2^f/f^ mice; W.X., Y.X., D.L., Y.L., W.X., and C.Y. conducted the experiments and interpreted data, and performed the statistical analysis; K.Y., Y.L., and G.M. were responsible for VTE patient blood samples and thrombus tissues collection; Y.L. kindly presented Myc‐pLVX‐SHP2 plasmids and some reagents; Y.K., K.Y., X.Z., H.C., and E.C. offered some constructive suggestions during the experimental process; W.X., Y.X., D.L., and X.Z. wrote and critically revised the manuscript and all authors approved of the final version of the manuscript.

## Supporting information

Supporting Information

## Data Availability

The data that support the findings of this study are available in the supplementary material of this article.
