# Supplementary material for: NanoSHP099‐Targeted SHP2 Inhibition Boosts Ly6Clow Monocytes/Macrophages Differentiation to Accelerate Thrombolysis
Source: Adv Sci (Weinh). 2024 Jan 21;11(13):2308166. doi: 10.1002/advs.202308166 (PMC10987109; doi:10.1002/advs.202308166)
Supplement: Supplementary file 1 — Supporting Information [file ADVS-11-2308166-s001.pdf]

ADVANCED SCIENCE
Open Access

Supporting Information

for *Adv. Sci.*, DOI 10.1002/advs.202308166

NanoSHP099-Targeted SHP2 Inhibition Boosts Ly6C^{low} Monocytes/Macrophages Differentiation to Accelerate Thrombolysis

Kejing Ying, Wanghao Xin, Yiming Xu, Dandan Lv, Huiqi Zhu, Yeping Li, Wangting Xu, Chao Yan, Yiqing Li, Hongqiang Cheng, Enguo Chen, Guofeng Ma, Xue Zhang* and Yuehai Ke**

In vivo assays

1. Mouse models and Patient tissue samples

C57BL/6 WT male mice were purchased from Hangsi Biological Inc., Hangzhou. $SHP2^{f/f}$ and LysmCre-$SHP2^{f/f}$ mice were generously provided by Professor Ke Yuehai. These mice were raised at Experimental Animal Center, Zhejiang University, which were subsequently used for breeding. Offspring were genotyped using standard PCR techniques with primers listed in Supplementary Table 3. SPF, 8-10 weeks old male mice were used for all experiments. All of the animal studies were conducted in accordance with the Guide for the Care and Use of Laboratory Animals of the National Institutes of Health and approved by the Institutional Animal Care and Use Committee of the Zhejiang University.

Thrombus tissue specimens were acquired from Department of Pathology, Run Run Shaw Hospital, Zhejiang University. Peripheral blood samples were freshly collected from VTE patients in Department of Respiratory Critical Care, Run Run Shaw Hospital, Zhejiang University, and healthy volunteers. The agreements were obtained from the patients with informed consent for the use of their specimens and physical examination results. All the human experiments were performed in compliance with the relevant ethical regulations, and approved by the local hospital Ethics Committee.

2. Inferior vena cava（IVC）stenosis model induced DVT model mice

The DVT model mice were induced as described previously.[1-3] Briefly, a midline laparotomy was performed and the bowel was moved to the side to expose the IVC. Side branches between the renal and iliac veins were ligated with 7/0 polypropylene suture (Jinhuan, Cat.HM702, Shanghai, China). A 5/0 nylon string (Jinhuan, Shanghai, China) was placed

parallel to the IVC. The IVC, along with the spacer string, were ligated tightly with a 7/0 polypropylene suture immediately below the level of the renal veins. Subsequently, the spacer string was removed allowing a little blood flow passing, and the mouse was closed in a layered fashion. All surgical procedures were performed under isoflurane (RWD, Shenzhen, China) anesthesia and proper thermal support was used to maintain body temperature.

The IVC stasis-induced DVT model of is highly reproducible and yields consistent thrombus starting from as early as a few hours, and extending to 10-20 days after stenosing. This model is extensively used to study the thrombus progression from acute to chronic inflammation within the thrombus. At euthanasia, the thrombosed vena cava immediately distal to the renal veins up to the iliac bifurcation, at early stage (day 1-3), middle stage (day 6-8) and late stage (day 11-13) after stenosing, was excised and used for histological and molecular analyses. Maximum thrombus formation is typically observed at day 3 post stenosis and thrombus resolution is typically measured by comparing thrombus weights between control and test animals at day 12 post- stenosis.

3. Lung metastatic tumor mouse models

The experimental tumor metastasis assay was performed in mice as described previously.[4, 5] In brief, B16-F10 melanoma cells (5×10^5 cells/mice) were resuspended in 100 μL PBS, and immediately injected into the tail veins of 8 weeks old C57BL/6 mice. Two days after implantation, mice were randomly divided into different groups subjected to various drugs treatments through tail intravenous injection till the endpoint. At the day 14 after B16-F10 cells implantation, mice were sacrificed, the presence of metastatic lesions on the lungs were evaluated by *ex vivo* imaging and histological analysis.

4. Systematic monocyte/macrophages depletion

To deplete monocyte/macrophages, 8 weeks old C57BL/6 mice were intravenously injected with Chlorophosphate liposomes (Yeasen, Cat.40337ES08, Shanghai, China) after DVT modeling and continued every third day at the indicated time points. Matching PBS liposomes (Yeasen, Cat.40338ES05, Shanghai, China) administration served as control. At the endpoint, mice were sacrificed and flow cytometry was used to verify the efficacy of monocyte/macrophages depletion. Data were analyzed using ACEA NovoExpree software.

5. SHP099 solution preparation and administration

SHP099 stock solution (30 mg/mL) was prepared by fully dissolving 20 mg SHP099 (Selleck, Cat.S6388, USA) powder in 333.33 μL DMSO. SHP099 storage solution was prepared by diluting stock solution in a mixed solvent of DMSO: PEG2000: PBS as 5:2:3, then the overall volume amounts to 666.66 μL. For administration of 30 mg SHP099 per kg mouse weight, and one 8 weeks old mouse approximately weighing 20 g, SHP099 solution was diluted as 6 mg/mL by PBS. By that analogy, SHP099 working solution was prepared as 3 mg/mL for middle-dose administration of 15 mg/kg, and 1.5 mg/mL for low-dose administration of 7.5 mg/kg. The solution dilution principle of intravenous free DiI is the same as that of Nanoshp099.

Mice were treated with either vehicle (mixed solvent of DMSO: PEG2000: PBS) or SHP099 solution (7.5, 15, 30 mg/kg) in a volume of 100 μL every other day via tail vein injections.

6. Immuno-histological staining and analysis

To assess the *in vivo* therapeutic potentials of SHP099 and NanoSHP099, mouse models were divided into different drugs treated groups including SHP099 in 7.5, 15, 30 mg/kg/2d, NanoSHP099 in 7.5, 15 mg/kg/2d, and vehicle (mixed solvent of DMSO: PEG2000: PBS) treated group. After the DVT model mice or lung metastatic tumor mouse models were established, the different formulations were injected via the tail vein as the arranged schedules. After therapy, venous thrombus tissues or lung tissues excised from mice were either embedded in OCT (Solarbio, Cat.4583, USA) and frozen, or fixed in 4% PFA (Beyotime, Cat.P0099, Shanghai, China) and embedded in paraffin, subsequently, tissue blocks were sectioned as 7 μm per slide.

To observe the thrombolytic effects in DVT mice, thrombus tissue sections were stained with Masson trichrome or Sirius Red (Sbjbio, Cat.BP-DL021, BP-DL027, Nanjing, China), and then scanned with Olympus VS200 digital scanner. The thrombus areas (mm^2) were calculated by 3.14 × (mean blood clot diameter/2)^2, after measuring thrombus diameter at cross-section. The collagen content percentages (%) were evaluated by the (collagen area/blood clot area) × 100%, analyzed with Image J software. To detect intra-thrombus monocyte/macrophages, paraffin-embedded sections fixed in 4% PFA and permeabilized by 0.25% Triton X-100 were stained with antibodies against CD115 (1:1000), CCR2 (1:500) and CX3CR1 (1:200) as listed in Supplementary Table 2. HRP-conjugated or Alexa Fluor antibodies (1:200) were used as secondary antibodies. Nuclei were counterstained using DAPI (Solarbio, Cat.D8200, USA). The staining was analyzed using a Nikon fluorescence microscope.

To observe the anti-tumor and anti-thrombus effects in lung metastatic tumor mouse models, the whole lung tissues were extracted integrally and sectioned sequentially to collect 20 slides of tissue sections at constant intervals. These sections were stained with H&E (Beyotime, Cat.C0105S, Shanghai, China), and adjacent sections were stained with Ki67 (1:1000) antibody (Abcam, Cat.ab15580, UK), following HRP-conjugated antibody (1:500) (Abmart, Cat.GU101019, Shanghai, China) used as secondary antibody. After scanned using Olympus VS200 digital scanner, the pulmonary vessels occluded with Eosin-stained blood clots were detected as pulmonary thromboembolism foci. For tumor measurement, the black neoplastic lesions (> 2 mm) on the surface of lung tissues, as well as the Ki67 positive foci in lung tissues sections were counted with Image J software.

7. Tissue single-cell suspensions preparation, FCM, FACS, and CyTOF assay

Tissue single-cell suspensions were prepared as previously reported.[6, 7] Lung, liver, kidney and spleen were excised and minced with fine scissors in PBS, and then placed in a rotating shake, after dispersed in RPMI medium containing 1 μg/mL collagenase I (Sigma-Aldrich, Cat.C5138, USA), for digestion 30 min at room temperature. Thrombus tissues were fully ground on ice with 1 mL syringe piston rod until the visible particles disappearing. These suspensions were filtered gently through a 70 μm cell strainer (BD Falcon, Cat.352350, USA), followed by 40 μm cell strainer, and then resuspended in 100 μL PBS.

FCM detection was performed similarly to previous description.[7, 8] Tissue single-cell suspensions were fluorescently stained with anti-mouse flow antibodies according to the experimental panels listed in supplementary Table 1. Cell debris and dead cells were excluded from the analysis based on scatter signals and Zombie Violet™ fluorescent staining

(BioLegend, Cat.423113, USA). Data were collected on ACEA NovoCyte analyzer and analyzed using NovoExpree software.

FACS was operated as previously reported,[9] briefly, $CD11B^{+}CD115^{+}Ly6G^{-}Ly6C^{high}$ and $CD11B^{+}CD115^{+}Ly6G^{-}Ly6C^{low}$ monocyte/macrophages subsets were respectively isolated from bone marrow single-cell suspension after stained with APC-CD11B, PE-CD115, FITC-Ly6G, PECy7-Ly6C flow antibodies and dead cell dye Zombie Violet™, by using the BD FACS Aria II, and gating was done using the BD FACSDiva™ software. Cell sorting was performed with 100 μm nozzle size and sorted directly into 15 mL tubes containing 6 ml of RPMI medium containing 20% FBS in order to minimize cellular stress. Cells (8×10^4) of each population of interest were sorted at a speed of 1500 cells/s.

Lung tissues single-cell suspensions acquired from Free-DiI or NanoSHP099 (with the same DiI concentration of 10 μg/mL) treated lung metastatic tumor mouse models were sorted by FCAS using BD FACS Aria II to respectively obtain $CD45^{+}$ immune infiltrating cells. The CyTOF assay to detect immune cells profiles was performed by PLTTECH Biological Inc.

8. Target ability detection of NanoSHP099

The targeting detection was performed as previous descriptions.[10-13] DiI-labeled NanoSHP099 liposomes or free-DiI solution (with the same DiI concentration of 10 μg/mL) were intravenously injected into mouse models at day 7 after IVC stenosis or tumor cells implantation. One day after drugs administration, the retroperitoneal vessels were exposed after a midline laparotomy when mice were under isoflurane anesthesia; subsequently, the DiI fluorescence images at the IVC thrombus lesions were captured via an *in vivo* imaging system (IVIS) Caliper IVIS Lumina II equipped with an excitation bandpass filter at 549 nm and

emission at 565 nm. Similarly, the DiI fluorescence intensities at thoracic and abdominopelvic areas of lung metastatic tumor mouse models were also captured and measured by the IVIS. After euthanasia of the mice, IVC thrombus, lung, and other major organs including heart, liver, spleen, and kidney were harvested to place on a black background; and the DiI fluorescence images and signal intensities of these *ex vivo* tissues were also detected at the laser of 549/565 nm.

Thrombus tissues acquired from NanoSHP099 or free-DiI treated-DVT mice, and lung tissues from NanoSHP099 treated-lung metastatic tumor mice were embedded in OCT and frozen, and subsequently sectioned as 7 μm slides in the dark conditions. After stained with DAPI, thrombus tissue sections were observed using a fluorescence confocal microscopy to compare the DiI fluorescence intensities between NanoSHP099 and free-DiI groups; and lung tissues sections were scanned to detect the location of NanoSHP099 in tumor and thrombus foci.

9. Mice IVC micro-CT scanning

After anesthesia, Iodoxanol contrast agent (HAIZHOU PHARMA, Cat. 92339-11-2, Zhejiang, China) was delivered into the different drugs treated-DVT model mice via micro-infusion pumps connected to the tail veins before micro-CT scanning was performed on a *in vivo* micro-CT device (Imalytics Preclinical).[14] Immediately, mice were scanned thoroughly at an isotropic nominal resolution of 18 μm, at 50 kV, 500 μA, with an exposure of 280 ms, and two-fold frame averaging. The collected data was processed using software provided by the manufacturer to export the representative scanning images in different dimensions.

10. IVC blood flow laser speckle imaging

The blood flow changes in the IVC thrombus lesions were measured by a laser speckle imaging system (RWD). This laser speckle imaging has been widely used to visualize relative patterns in vessel blood flow of different parts without contact *in vivo* animal models.[15, 16] This device simply includes a laser for illuminating the tissue surface and a camera for detecting the back-scattered light. DVT mice after vehicle, SHP099 and NanoSHP099 treatments were fixed on the worktop under isoflurane anesthesia. The IVC vessels were fully exposed after laparotomy operation with a near-infrared laser diode (808 nm, 4.5 mW) illuminating at the same positions to acquire blood flow images at high-spatial resolution and record the blood flow changes. The sham mice were performed as the same procedure without modeling or drugs treatments ahead to set the normal IVC blood flow control.

11. Bio-compatibility evaluation

The *in vivo* safety evaluation of nanomedicine included tissue organ structures[8, 10, 11, 13] and serum biochemical indices.[17, 18] Primarily, heart, liver, spleen, lung and kidney excised from various SHP099 formulations treated DVT model mice were performed H&E histological staining to observe the pathological changes. Meanwhile, the serum biochemical indices associated to functions of liver, kidney, and inflammation and fibrinolytic systems, including ALT, AST, BUN, CREA, CRP, and FDP were tested by an animal biochemistry analyzer conducted in Pinuofei Biological Inc.

In vitro assays

1. Cell isolation and Cell culture

Mice lung micro-vessel endothelial cells, monocyte/macrophages, neutrophils, and fibroblasts were isolated as previous descriptions.[19-22] B16-F10, HEK293T, Raw 264.7 were purchased from Procell Biological Inc.

Cell line B16-F10, HEK293T, and primary fibroblasts were maintained at 37 °C in DMEM added with 10% fetal FBS and 1% PSG. Raw 264.7, primary neutrophils and monocyte/macrophages were cultured in DMEM containing 10% inactivated FBS and 1% PSG at 37 °C in a humidified atmosphere containing 5% CO_2 at 37 °C. Primary mice ECs were cultured in endothelial basal medium supplemented with growth supplements at 37 °C in 5% CO_2 atmosphere.

2. RNA-seq analysis and qPCR

Ly6C^{high} and Ly6C^{low} monocyte/macrophages groups were simultaneously isolated from bone marrow cell suspensions by FACS, and three biological replicates for each subpopulation group. Total RNA of each group was extracted using RNA extraction Kit (Axygen, Cat.AP-MN-MS-RNA-50, USA), in accordance with the manufacturer's instruction. RNA sequence was performed by Biomarker Biological Inc.

After samples returned, total RNA was reversed transcribed using qPCR RT Kit (TOYOBO, Cat.FSQ-101, Japan) according to the manufacturer's instruction, and then amplified for 40 cycles, 15 seconds at 95 ˚C and 60 seconds at 60 ˚C using UltraSYBR Mixture (CWBIO, Cat.CW0957S, Jiangsu, China). The expressions of genes associated to collagen synthesis and degradation were determined relative to the expression of the actin, used as an internal control. The primers sequences used for real-time PCR are listed in supplemental Table 3.

3. Plasmid or siRNA transfection, Co-IP, and Protein spectrum identification

HEK293T cells were seeded in the 6-well plate to reach 70% confluency. The transfection compounds of plasmids (SHP2-Myc, NR4Al-Flag, C/EBPβ-HA) or siRNA (siC/EBPβ), mixed with Lipo3000 (Invitrogen, Cat.L3000-015, USA), prepared in accordance with the manufacturer's instruction, were gently added to cell culture medium and mixed. After 6 h of culture in 37 ° C medium, the old medium was discarded and replaced with a fresh complete medium containing PSG.

At the scheduled time points, cells were lysed in IP lysis buffer, and 10% protein lysates were used as input. Then, antibodies against either SHP2 (CST, Cat.3397, USA) or IgG (Beyotime, Cat.A7001, Shanghai, China) were incubated together with protein A Sepharose (BIO-RAD, Cat.1614013, USA) for 20 min at room temperature under rotation. After washing with 0.1% PBST, protein A Sepharose-antibodies complexes were incubated together with the remaining 90% cell extracts overnight at 4 °C. Next day, unbound proteins were removed by washing with 0.1% PBST, Sepharose-antibodies-proteins complexes were separated via SDS-PAGE followed by western blotting on a BioTrace™ NC membrane (PALL, Cat.52845922, USA). Proteins were visualized by an Odyssey two-color infrared imaging system (LI-COR) after treated with IRDye 680LT/IRDye 800CW secondary Abs (LI-COR, Lincoln, NE).

Similar to the previous operations,[23, 24] proteins immunoprecipitated by IgG isotype and anti-NR4A1 antibodies (Abmart, Cat.TD7850, Shanghai, China) in NR4A1 overexpressed HEK293T cells after NR4Al-Flag plasmids transfections, and proteins immunoprecipitated by IgG isotype and anti- C/EBPβ antibodies (Abmart, Cat.T55276, Shanghai, China) in C/EBPβ overexpressed HEK293T cells after C/EBPβ-HA plasmids transfections. The protein mass spectrum identification of theses enriched protein samples in three biological repeats separated

in SDS-PAGE gels was performed by Abace Biological Inc.

4. Immunocytochemistry

HEK293T cells with over-expressions of SHP2 and NR4A1, or SHP2 and C/EBPβ, were fixed with 4% PFA for 20 min and permeabilized with 0.1% Triton X-100 for 5 min, followed by blocking in 10% goat serum for 1 h at room temperature. Then, the cells were probed at 4 °C overnight with anti-SHP2, anti-pSHP2 (Abcam, Cat.ab62322, USA), and anti-NR4A1 or anti-C/EBPβ antibodies listed in Supplementary Table 2, and then incubated with goat anti-rabbit IgG H&L (Alexa Fluor 594, 1:200, ab150084, Abcam) and goat anti-mouse IgG H&L (Alexa Fluor 488, 1:200, ab150117, Abcam) for 1 h at room temperature. Nuclei were stained with DAPI, and the cells were imaged using a confocal laser scanning microscope.

Thrombotic tissues from DVT mice after Chlorophosphate liposomes (n=1) or PBS (n=1) treatments. were fixed with 4% PFA for 20 min and permeabilized with 0.1% Triton X-100 for 5 min, followed by blocking in 10% goat serum for 1 h at room temperature. Then, the cells were probed at 4 °C overnight with anti-CD115 (CST Cat.3155S, USA), and then incubated with goat anti-rabbit IgG H&L (Alexa Fluor 594, 1:200, ab150084, Abcam) for 1 h at room temperature and then with the DAB Horseradish Peroxidase Color Development Kit (Beyotime, Cat. P0202, Shanghai, China). And the cells were imaged using a confocal laser scanning microscope and select five 40× field of view to analyze. Cells with ≤25% staining were scored as (1); cells with 26-50% staining were scored as (2); cells with 51-75% staining were scored as (3); and cells with 76-100% staining were scored as (4). The staining color was scored as light-yellow particle (1), brown-yellow particle (2), and brown particle (3). The final score was defined as staining number score multiplied by staining color score (12). The scores

of negative expression were between 0 and 5, and the scores that exceeded 5 were identified as positive expression.

5. Western blot

After stimulations of Raw264.7 with IL6 (10 ng/mL), the cultured cells were extracted by RIPA lysis buffer. After concentration measurement with BCA assay ((Beyotime, Cat.P0010, Shanghai, China)), proteins mixed with loading buffer, were heated to denature and separated by SDS-PAGE on 10% polyacrylamide gels. After transferred to NC membranes, the membranes were blocked with TBST containing 5% milk for 1 hour at room temperature. Subsequently, the membranes were incubated with primary Abs at 4 °C overnight; then washed by TBST three times and treated with IRDye 680LT/IRDye 800CW secondary Abs (LI-COR, Lincoln, NE) for 1 hour at room temperature. Finally, the signals were detected by an Odyssey two-color infrared imaging system.

6. CCL2, CX3CL1 ELISA Assays

Blood samples acquired from SHP099 or vehicle treated DVT mice by heart punctures at scheduled time point, was collected into tubes freshly pretreated with 1% heparin (Sigma-Aldrich, Cat.H3149, USA) and centrifuged at 3000 rpm for 10 min at 4 °C to obtain plasma. Meanwhile, thrombus tissues obtained from variously treated DVT mice were milled in equal volumes of PBS on ice, and then filtered through 40 μm cell filters to collect thrombus lysis fluids. The levels of monocyte chemokine CCL2, CX3CL1 in plasma and thrombus lysis samples were measured with mice CCL2 ELISA kit (Elabscience, Cat.E-EL-M0006c, Wuhan, China) and CX3CL1 ELISA kit (Elabscience, Cat.E-EL-M0267c, Wuhan, China) according to manufacturer’s instruction.

7. Dual-luciferase reporter system

The Luciferase reporter plasmids NR4A1-promoter-pGL3-Basic (with Firefly Luciferase vector) and pRL-TK (with Renilla Luciferase vector) were purchased from Fenghui Bioscience Inc (Changsha, China). These plasmids were amplified, followed by transformation of DH5α competent cells cultured in Amp^+ LB plates overnight. After shaking the DH5α competent cells in LB medium for 12–16 h. The plasmid was extracted and stored at -80 °C. HEK-293T cells were seeded into 24-well plates and cultured until they reached a confluency of approximately 70%; then, the siC/EBPβ or C/EBPβ-HA plasmids were transfected into different groups. After 12 h, luciferase reporter plasmid, NR4A1-promoter-pGL3-Basic and pRL-TK control were co-transfected into HEK-293T cells. After 24 h, a dual luciferase reporter assay[25] was performed using a dual luciferase reporter system (Promega, Cat.TM040, USA), following the manufacturer's instruction. The firefly luciferase activity was normalized to Renilla luciferase activity for quantification.

8. Synthesis of CREKA-DiI-Lipo@SHP099

CREKA-DiI-Lipo@SHP099 were prepared similarly to previous description of other drug nanoliposomes.[13, 26, 27] The phospholipid material DSPE-PEG-MAL (Ruixi Bio, Cat.LP-R4-136, Xi'an, China) possess both hydrophilicity and hydrophobicity, can self-assemble into spherical particles in aqueous solution. Meanwhile, the hydrophobic small molecule drug SHP099 added into the solution could be wrapped in the hydrophobic area of phospholipid spheres dispensing with chemical bonding. Then, DSPE-PEG-MAL was connected to CREKA (Cys-Arg-Glu-Lys-ALA) (Qiyue Bio, Cat.Q-0093597, Xi'an, China) by a thioether linkage

between the MAL in DSPE-PEG-MAL and the sulfhydryl groups in CREKA peptide, and the liposomes were labeled fluorescence by staining the liposoluble DiI dyes (Meilunbio, Cat.MB4240, Dalian, China).

In detail, (1) CREKA was conjugated via thioether linkage to DSPE-PEG2000-MAL (molar ratio 1:1) in a water/methanol solution (90:10, v/v) reacted at room temperature for 6 h. The DSPE-PEG2000-CREKA compound was purified by dialysis tubing to remove residual CREKA. (2) Lipid nanovesicles were prepared via thin film hydration method. HSPC/cholesterol /DiI/SHP099 at the required ratios were fully dissolved in 3 mL CHCL3 solvent, and a lipid film was formed by the removal of the organic solvents with rotary evaporation, and then added DSPE-PEG2000-CREKA, hydrated with PBS buffer (pH 7.4) at 40 °C for 1 h. The resulting nanovesicles were sonicated at 4 °C for 30 min and subsequently extruded through 200 nm polycarbonate membranes (Avanti, 610000-1EA, USA) to obtain homogenized nanoparticles. (3) The dialysis process was utilized to remove the unencapsulated SHP099 and DSPE-PEG2000-CREKA, with a 50nm polycarbonate membrane (Ruixibio, Cat.R-Y-LZ-0973, Xi’an, China).

9. Characterization of CREKA-DiI-Lipo@SHP099

The hydrodynamic size and Zeta potential of the nanoparticles were measured by dynamic light scattering (DLS, ZetaSizer Nano ZS90, Malvern Instruments, USA) at a concentration of 0.018 mg/mL after diluted 100 times with PBS. The particle size of the NanoSHP099 in PBS, and PBS containing 20% FBS was monitored by DLS over two and a half days to evaluate the stability of the nanoparticles.

The morphology of NanoSHP099 was visualized by a scanning electron microscope (SEM; Thermo FEI, Nova Nano 450, Czech) at an accelerating voltage of 5 kV, after sputter-coated

with a layer of platinum in a sputter-coating chamber (Leica, EM ACE600, Germany).

The fluorescence intensities of NanoSHP099 in PBS buffer (pH 7.4) were performed by a microplate reader equipped with a 549/565 nm laser (Molecular Devices, Id5, USA).

The amounts of SHP099 encapsulated in the nanoliposomes were determined by the chemical spectrophotometry assay. The encapsulation efficiency (EE) was calculated by the following equation $\%EE=(m_i/m_t) \times 100\%$, where m_i is the weight of encapsulated SHP099 and m_t is the total initial weight of SHP099 in the loading solution. Firstly, a series of concentrations (1. 0.5, 0.25, 0.125, 0.0625, 0.0312, 0.0156, 0 mg/mL) of free SHP099 diluted solutions were prepared, and the OD values of samples at 280/400 nm UV were determined, to draw a standard curve. Then, NanoSHP099 liposomes were incubated with Triton X-100 at 1:1 volume for membrane rupture releasing encapsulated SHP099. After centrifuged at 4 °C, 12000 g, 30 min to deposit lipid membranes and filtrated through a 0.45 μm filter, the concentration of SHP099 in collected supernatant was measured according to above standard curve. In our study, the concentration of SHP099 in NanoSHP099 measured as above description was 1.08 mg/mL. A total of 10 mLNanoSHP099 was prepared, and the total weight of SHP099 drug powder consumed in the preparation process was 13 mg. Therefore, the drug encapsulation rate of SHP099 in NanoSHP099 is 83%. The drug loading efficiency (DL %) was calculated according to the following formula: DL%=(Amount of drug loaded in NPs)/(NPs weight) × 100% = 5.3%.

In vitro release kinetics of SHP099 from NanoSHP099 comparing to free SHP099 were studied by a dialysis method. 1 mL of SHP099 solution and 1 mL of NanoSHP099 suspension (with equal SHP099 concentration of 1.08 mg/mL) were respectively sealed in two same dialysis bags with a molecular weight cutoff of 3.5 kDa and respectively immersed in two

beakers of 50 mL FBS containing of 1% Tween 80 (pH 7.4) under gentle shaking at 37 °C. At certain intervals (1, 2, 4, 6, 8, 10, 12, 24, and 48 h), 0.2 mL of the solutions outside the bags were collected to determine the released SHP099 amount by above-described chemical spectrophotometer assay with a UV detector measuring absorbance at 280/400 nm. Equal volumes of pre-warmed fresh FBS solutions were supplemented to the release media.

10. Cytotoxicity, Proliferation, Apoptosis, and Cell cycle detections

The cytotoxicity of NanoSHP099 detected by CCK8 assay was similar to previous description.[14] In brief, 100 μL Raw264.7 cell suspension was prepared in a 96-well plate and incubated in an incubator (37 °C, 5% CO_2) overnight. After respectively treated by vehicle, and equal concentrations (10 μM) of SHP099 or NanoSHP099 for 24 h, 10 μL CCK8 solution (DOJINDO, Cat.CK04, Japan) per well was added and the culture plate continued to be incubated in the incubator for 2 h. The absorbance at 450 nm was measured with a microplate reader at end-point time.

To detect the proliferation influence of NanoSHP099, equal numbers (5×10^4) of Raw264.7 cells were stained with CFSE (Beyotime, Cat.C0051, Shanghai, China) according to manufacturer's instruction, and then added to different culture dishes. After above-mentioned various treatments, these dishes were subsequently incubated in 37 °C, 5% CO_2. Every day for the next three days, 200 μL cell suspension each group was obtained from digested Raw264.7 cell suspensions (the remaining cell suspensions continued to be cultured for the next measurement), and then measured by FCM to detect their fluorescence intensities at FITC channel.

Equal numbers (1×10^5) of Raw264.7 cells, treated by vehicle, SHP099 (10 μM) or

NanoSHP099 (10 μM) for 24 h, were acquired to be stained with Annexin V and PI dyes as the apoptosis detection kit's instruction (Yeasen, Cat.40302ES20, Shanghai, China). The apoptotic cells (Annexin V^+ PI^+) were detected by ACEA flow cytometer at FITC and PE dual-fluorescent channels.

As for cell cycle measurement, prepared cell suspensions after above treatments, were fixed in 70% precooled ethanol for 2 h and then washed with PBS. After filtered through 40 μL cell filters, cells were suspended in RNase A solution (Yeasen, Cat.40301ES50, Shanghai, China) and incubated at 37 °C for 30 min. After stained with PI fluorescence, cells were detected by ACEA flow cytometer at PE channel, and then analyzed using NovoExpree software to identify cell cycle distribution.

11. *In vitro* phagocytic ability detection

The abilities of different types of primary cells to phagocytose NanoSHP099 were evaluated by *in vitro* co-culture experiment.[28] Pulmonary vascular endothelial cells, fibroblasts, neutrophils and monocyte/macrophages respectively resuspended in 1 mL cell suspensions were seeded in culture plates preplaced with cell slides. When these cells grow to the same cell confluences, approximately 70%, equal volumes (10 μL) of NanoSHP099 were added into cell culture plates. After 1 hour incubation in dark, cells media were discarded and cells were washed three times with PBS. After stained with Alexa Fluor 488-conjugated phalloidin (Themro FEI, Cat.R37110, USA) marking cytoskeleton, and with DAPI staining Nuclei, these cells were imaged using a confocal laser scanning microscope. The intracellular NanoSHPP099 particles were counted with Image J software.

12. Gelatin zymography assay

The gelatin zymography assay was performed similarly as previously reported.[2, 29] Firstly, protein concentrations in Ly6C monocyte/macrophage subsets or thrombus lysis samples were determined by BCA assays, and adjusted to the same protein concentration. As the kit instruction (Real-times, Cat.RTD6143, Beijing, China) indicated, after add 5 × loading buffer (without β-mercaptoethanol or DTT) to samples, per sample contains 20 μg protein was loaded into 7% polyacrylamide gel containing 1% gelatin. After a good separation strip in gel is obtained under running at 150 V, the gel was incubated in A buffer for 1 h to restore collagenase activity, and then immersed in B buffer containing cofactors necessary for gelatinase reaction at room temperature for 15 h. Subsequently, the gel was incubated in FastBlue protein staining solution for 1 h, followed by ddH_2O wash to appear transparent bands.

Data Sharing Statement

For original data, please contact xinwanghao86@163.com

References:

[1] NOSAKA M, ISHIDA Y, KIMURA A, KUNINAKA Y, INUI M, MUKAIDA N, KONDO T. Absence of IFN-gamma accelerates thrombus resolution through enhanced MMP-9 and VEGF expression in mice [J]. J Clin Invest, 2011, 121(7): 2911-20.

[2] MUKHOPADHYAY S, ANTALIS T M, NGUYEN K P, HOOFNAGLE M H, SARKAR R. Myeloid p53 regulates macrophage polarization and venous thrombus resolution by inflammatory vascular remodeling in mice [J]. Blood, 2017, 129(24): 3245-55.

[3] WANG L, WANG P, ZHANG F, GUO X, CHEN X, GUAN D, ZHAO R. Mouse inferior vena cava stenosis model with all branches interrupted may help deep vein thrombosis research [J]. J Thromb Haemost, 2023, 21(7): 1995-7.

[4] ZHU Z, HUANG J, LI X, XING J, CHEN Q, LIU R, HUA F, QIU Z, SONG Y, BAI C, MO Y Y, ZHANG Z. Gut microbiota regulate tumor metastasis via circRNA/miRNA networks [J]. Gut Microbes, 2020, 12(1): 1788891.

[5] KADIOGLU O, SAEED M E M, MAHMOUD N, HUSSEIN AZAWI S S, RINCIC M, LIEHR T, EFFERTH T. Identification of metastasis-related genes by genomic and transcriptomic studies in murine melanoma [J]. Life Sci, 2021, 267: 118922.

[6] HU D, YIN C, MOHANTA S K, WEBER C, HABENICHT A J. Preparation of Single Cell Suspensions from Mouse Aorta

[J]. Bio Protoc, 2016, 6(11).
[7] REICHARD A, ASOSINGH K. Best Practices for Preparing a Single Cell Suspension from Solid Tissues for Flow Cytometry [J]. Cytometry A, 2019, 95(2): 219-26.
[8] DENORME F, MARTINOD K, VANDENBULCKE A, DENIS C V, LENTING P J, DECKMYN H, VANHOORELBEKE K, DE MEYER S F. The von Willebrand Factor A1 domain mediates thromboinflammation, aggravating ischemic stroke outcome in mice [J]. Haematologica, 2021, 106(3): 819-28.
[9] CREMERS N A J, VAN DEN BOSCH M H J, VAN DALEN S, DI CEGLIE I, ASCONE G, VAN DE LOO F, KOENDERS M, VAN DER KRAAN P, SLOETJES A, VOGL T, ROTH J, GEVEN E J W, BLOM A B, VAN LENT P. S100A8/A9 increases the mobilization of pro-inflammatory Ly6C(high) monocytes to the synovium during experimental osteoarthritis [J]. Arthritis Res Ther, 2017, 19(1): 217.
[10] KANG C, GWON S, SONG C, KANG P M, PARK S C, JEON J, HWANG D W, LEE D. Fibrin-Targeted and H(2)O(2)-Responsive Nanoparticles as a Theranostics for Thrombosed Vessels [J]. ACS Nano, 2017, 11(6): 6194-203.
[11] ZHAO Y, XIE R, YODSANIT N, YE M, WANG Y, GONG S. Biomimetic fibrin-targeted and H(2)O(2)-responsive nanocarriers for thrombus therapy [J]. Nano Today, 2020, 35.
[12] CHANG L H, CHUANG E Y, CHENG T M, LIN C, SHIH C M, WU A T, JHENG P R, LU H Y, SHIH C C, MI F L. Thrombus-specific theranostic nanocomposite for codelivery of thrombolytic drug, algae-derived anticoagulant and NIR fluorescent contrast agent [J]. Acta Biomater, 2021, 134: 686-701.
[13] SHI L, WANG Y, WANG Q, JIANG Z, REN L, YAN Y, LIU Z, WAN J, HUANG L, CEN B, HAN W, WANG H. Transforming a toxic drug into an efficacious nanomedicine using a lipoprodrug strategy for the treatment of patient-derived melanoma xenografts [J]. J Control Release, 2020, 324: 289-302.
[14] GROVER S P, SAHA P, JENKINS J, MUKKAVILLI A, LYONS O T, PATEL A S, SUNASSEE K, MODARAI B, SMITH A. Quantification of experimental venous thrombus resolution by longitudinal nanogold-enhanced micro-computed tomography [J]. Thromb Res, 2015, 136(6): 1285-90.
[15] XU C, TING W, TENG Y, LONG X, WANG X. Laser Speckle Contrast Imaging for the Objective Assessment of Blood Perfusion in Keloids Treated With Dual-Wavelength Laser Therapy [J]. Dermatol Surg, 2021, 47(4): e117-e21.
[16] KIKUCHI S, MIYAKE K, TADA Y, UCHIDA D, KOYA A, SAITO Y, OHURA T, AZUMA N. Laser speckle flowgraphy can also be used to show dynamic changes in the blood flow of the skin of the foot after surgical revascularization [J]. Vascular, 2019, 27(3): 242-51.
[17] ANSAR S, ALSHEHRI S M, ABUDAWOOD M, HAMED S S, AHAMAD T. Antioxidant and hepatoprotective role of selenium against silver nanoparticles [J]. Int J Nanomedicine, 2017, 12: 7789-97.
[18] FENG L, YANG X, SHI Y, LIANG S, ZHAO T, DUAN J, SUN Z. Co-exposure subacute toxicity of silica nanoparticles and lead acetate on cardiovascular system [J]. Int J Nanomedicine, 2018, 13: 7819-34.
[19] WANG J, NIU N, XU S, JIN Z G. A simple protocol for isolating mouse lung endothelial cells [J]. Sci Rep, 2019, 9(1): 1458.
[20] WEISCHENFELDT J, PORSE B. Bone Marrow-Derived Macrophages (BMM): Isolation and Applications [J]. CSH Protoc, 2008, 2008: pdb prot5080.
[21] SWAMYDAS M, LUO Y, DORF M E, LIONAKIS M S. Isolation of Mouse Neutrophils [J]. Curr Protoc Immunol, 2015, 110: 3 20 1-3 15.
[22] XU J. Preparation, culture, and immortalization of mouse embryonic fibroblasts [J]. Curr Protoc Mol Biol, 2005, Chapter 28: Unit 28 1.
[23] KAULICH P T, CASSIDY L, WEIDENBACH K, SCHMITZ R A, THOLEY A. Complementarity of Different SDS-PAGE Gel Staining Methods for the Identification of Short Open Reading Frame-Encoded Peptides [J]. Proteomics, 2020, 20(19-20): e2000084.
[24] CRAMER R, SAXTON M, BARNOUIN K. Sample preparation of gel electrophoretically separated protein binding partners for analysis by mass spectrometry [J]. Methods Mol Biol, 2004, 261: 499-510.
[25] WANG Y, REN X, LI W, CAO R, LIU S, JIANG L, CHENG B, XIA J. SPDEF suppresses head and neck squamous cell carcinoma progression by transcriptionally activating NR4A1 [J]. Int J Oral Sci, 2021, 13(1): 33.
[26] PALASSI S, VALIZADEH H, ALLAHYARI S, ZAKERI-MILANI P. Preparation and In Vitro Characterization of

Enoxaparin Nano-liposomes through Different Methods [J]. Adv Pharm Bull, 2021, 11(2): 295-300.
[27] AL-AMIN M D, BELLATO F, MASTROTTO F, GAROFALO M, MALFANTI A, SALMASO S, CALICETI P. Dexamethasone Loaded Liposomes by Thin-Film Hydration and Microfluidic Procedures: Formulation Challenges [J]. Int J Mol Sci, 2020, 21(5).
[28] HUI Y, YI X, WIBOWO D, YANG G, MIDDELBERG A P J, GAO H, ZHAO C X. Nanoparticle elasticity regulates phagocytosis and cancer cell uptake [J]. Sci Adv, 2020, 6(16): eaaz4316.
[29] RAYKIN J, SNIDER E, BHERI S, MULVIHILL J, ETHIER C R. A modified gelatin zymography technique incorporating total protein normalization [J]. Anal Biochem, 2017, 521: 8-10.

FIGURE LEGENDS

Schematic diagram:

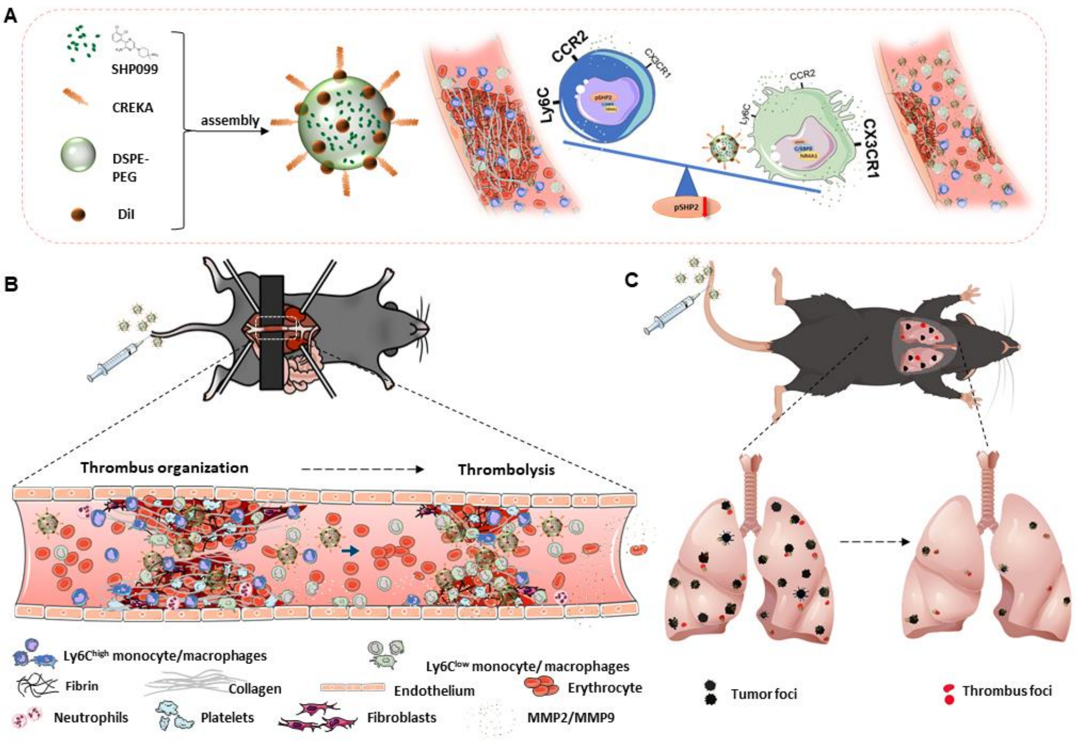

Scheme 1.

A. Schematic illustration of the components of CREKA-DiI-Lipo@SHP099 (NanoSHP099), and it serves as a potent thrombus-targeting thrombolytic agent by specifically promoting Ly6C^{low} monocyte/macrophages differentiation with SHP2 activity inhibition.

B. In DVT modeling mice, NanoSHP099 tail vein injection promote venous thrombus resolution with increasing Ly6C^{low} monocytes/macrophages infiltration highly expressing MMP2/9.

C. In lung metastatic tumor model mice, intravenously injected NanoSHP099 are enriched in lung tissues, bi-directionally targeting tumor and thrombotic lesions, and exerts the dual-roles of anti-tumor and anti-thrombus.

FigS1.

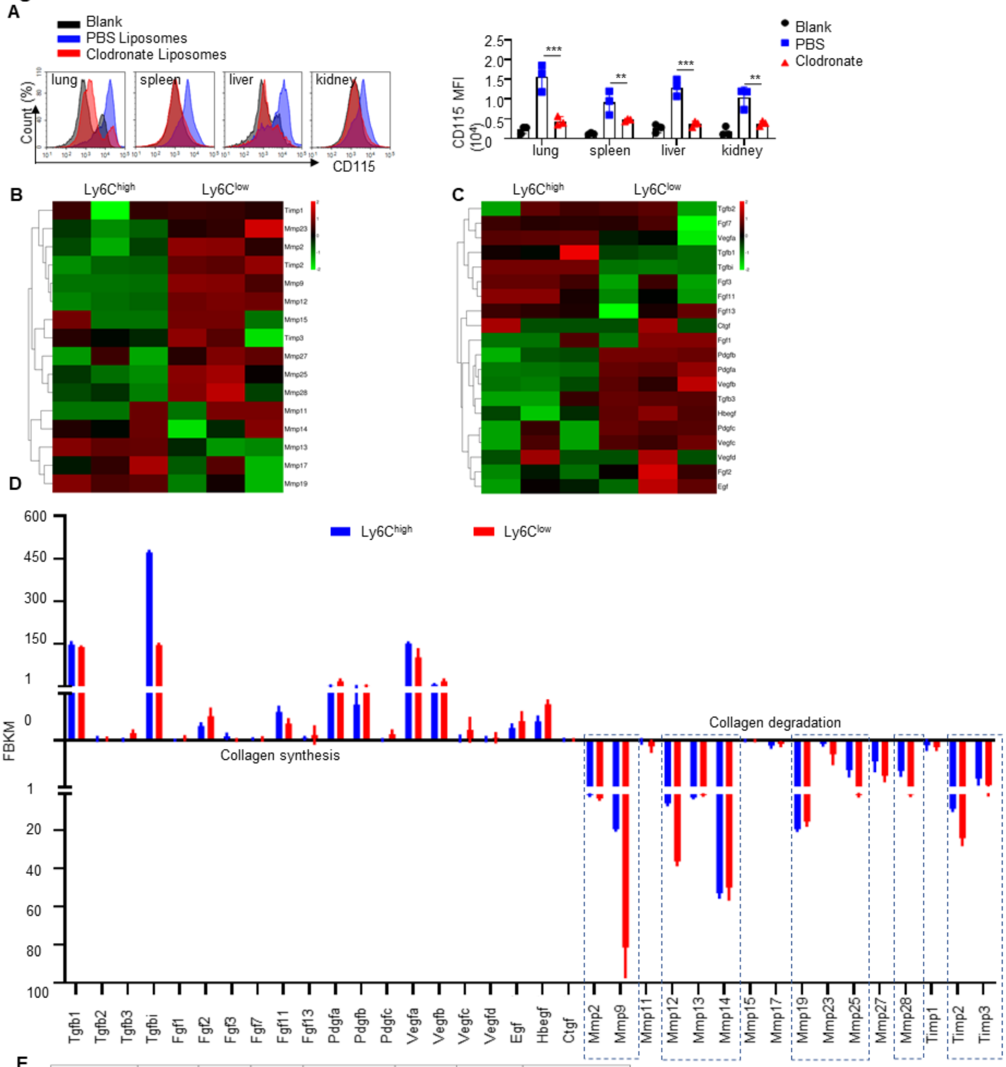

E

Standard (mg/ml)	Absorbance			Sample number	Absorbance	Dilution ratio	Protein Concentration
0.5	1.001			1 $Ly6c^{high}$	0.17	100	3.875
0.4	0.755			2 $Ly6c^{high}$	0.167	100	3.702
0.3	0.517	delete		3 $Ly6c^{high}$	0.174	100	4.105
0.2	0.452			4 $Ly6^{low}$	0.156	100	3.070
0.1	0.301			5 $Ly6^{low}$	0.174	100	4.105
0.05	0.203			6 $Ly6^{low}$	0.161	100	3.357
0.025	0.13			32SHP2$^{f/f}$Lysm cre	2.035	5	5.554
0	0.094			33SHP2$^{f/f}$Lysm cre	1.811	5	4.911
				35SHP2$^{f/f}$Lysm cre	2.132	5	5.833
				34SHP2$^{f/f}$	2.373	5	6.526
				36SHP2$^{f/f}$	2.472	5	6.811
				37SHP2$^{f/f}$	2.523	5	6.957
				1.1control	3.075	5	8.544
				1.2control	2.949	5	8.182
				1.3control	3.399	5	9.475
				1.4SHP099	3.07	5	8.529
				1.5SHP099	3.289	5	9.159
				1.6SHP099	2.933	5	8.136
				1.7NanoSHP099	3.317	5	9.239
				1.8NanoSHP099	3.405	5	9.492
				1.9NanoSHP099	3.336	5	9.294

Supplementary Figure 1.

A. Above: The overlaying peaks of $CD115^{+}$ monocytes/macrophages in lung, spleen, liver, and kidney tissue cell suspensions acquired from DVT mice after various treatments of vehicle, PBS liposomes, and Clodronate liposomes.

   Below: The statistics data of mean fluorescence intensities of CD115 in various tissues suspensions analyzed by FCM.

B. Cluster analysis heatmaps showing collagen synthesis related growth factors related MMPs differentially expressed in $Ly6C^{high}$ and $Ly6C^{low}$ monocytes/macrophages.

C. Cluster analysis heatmaps showing collagen synthesis related collagen degradation related MMPs differentially expressed in $Ly6C^{high}$ and $Ly6C^{low}$ monocytes/macrophages.

D. The quantitative analysis of gene expression levels of collagen synthesis related growth factors and collagen degradation related MMPs in RNA-seq.

E. Protein Quantitative Data of BCA Method in Gelatin zymography assay. Fig.1I（Red background）、Fig. 2G（Yellow background）、Fig. 7F（Blue background）

FigS2.

A

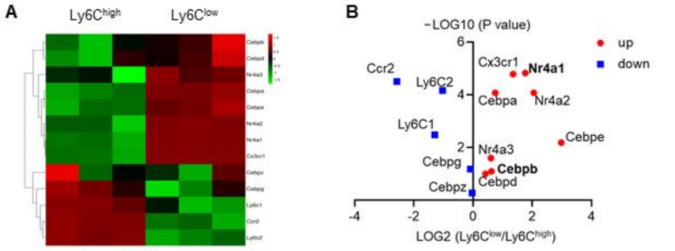

C

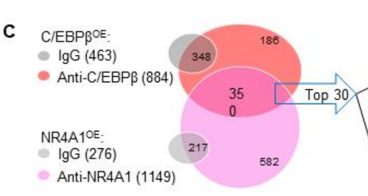

C/EBPβ ip

No.	Gene name	Score	Idensity
1	ACTG1	220.9	1.18E+10
2	EEF1A1	126.91	6.85E+09
3	HIST1H1D	80.55	5.54E+09
4	RPLP0	117.08	1.15E+09
5	RPL6	103.2	1.09E+09
6	RPL7A	71.694	1.09E+09
7	PTPN11	109.71	9.29E+08
8	PRDX1	37.013	7.98E+08
9	RPL12	24.134	5.74E+08
10	CPNE3	66.604	5.13E+08
11	RPL26	40.254	5.12E+08
12	HIST1H1C	10.246	4.80E+08
13	ANXA2	141.9	4.34E+08
14	FBLL1	6.4446	3.08E+08
15	RPL15	50.297	2.58E+08
16	HNRNPH1	89.875	2.49E+08
17	RPS10	34.239	2.34E+08
18	Ddx3x	80.505	2.27E+08
19	CORO1C	65.776	2.11E+08
20	RPL19	24.814	1.96E+08
21	GATAD1	57.196	1.94E+08
22	DDX39A	51.501	1.90E+08
23	TADA2A	64.627	1.89E+08
24	HIST1H1E	7.8056	1.81E+08
25	PRPF19	52.323	1.79E+08
26	PHGDH	39.426	1.69E+08
27	ZNF146	36.951	1.49E+08
28	ASF1A	27.134	1.46E+08
29	RPS25	19.328	1.43E+08
30	PCNA	61.543	1.37E+08

NR4A1 ip

No.	Gene name	Score	Idensity
1	ACTB	323.31	3.56E+10
2	HIST1H1C	33.056	6.51E+09
3	EEF1A1	118.48	6.48E+09
4	RPL6	113.34	4.44E+09
5	RPL7A	37.979	2.41E+09
6	RPLP0	112.06	2.16E+09
7	DOCK7	44.615	2.11E+09
8	RPL12	37.726	1.36E+09
9	RPL26L1	103.87	1.26E+09
10	ANXA2	93.058	1.20E+09
11	CPNE3	57.651	9.43E+08
12	DDX3X	65.624	8.19E+08
13	RPL15	78.598	6.86E+08
14	RPL19	9.726	6.45E+08
15	PRDX1	6.3277	5.87E+08
16	CKMT1A	29.327	4.26E+08
17	LIMA1	45.895	3.80E+08
18	RPL26	1.3153	3.74E+08
19	AMOTL1	18.559	3.54E+08
20	HNRNPH1	62.473	3.40E+08
21	VAPA	26.221	3.28E+08
22	CORO1C	38.254	3.14E+08
23	RPS10	2.7838	3.10E+08
24	RPLP1	12.487	2.89E+08
25	FBLL1	1.999	2.62E+08
26	U2af2	9.2924	2.60E+08
27	PTPN11	13.762	2.56E+08
28	MKI67	7.2495	2.29E+08
29	NAP1L1	12.176	2.27E+08
30	GNB1	5.2178	2.09E+08

D

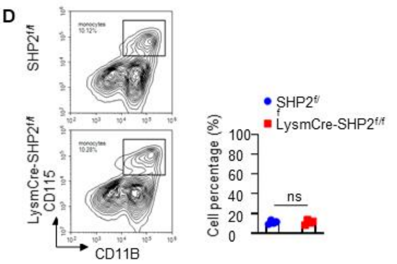

E

F

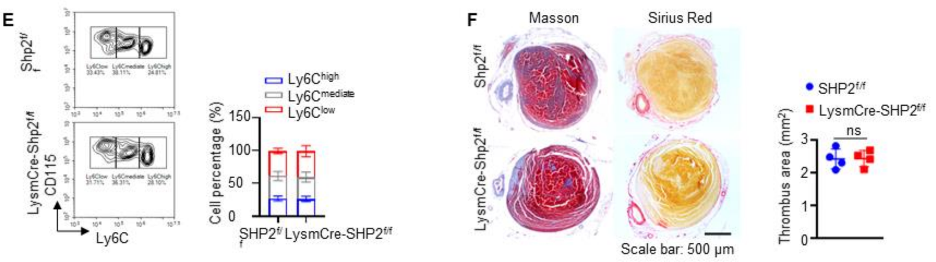

G

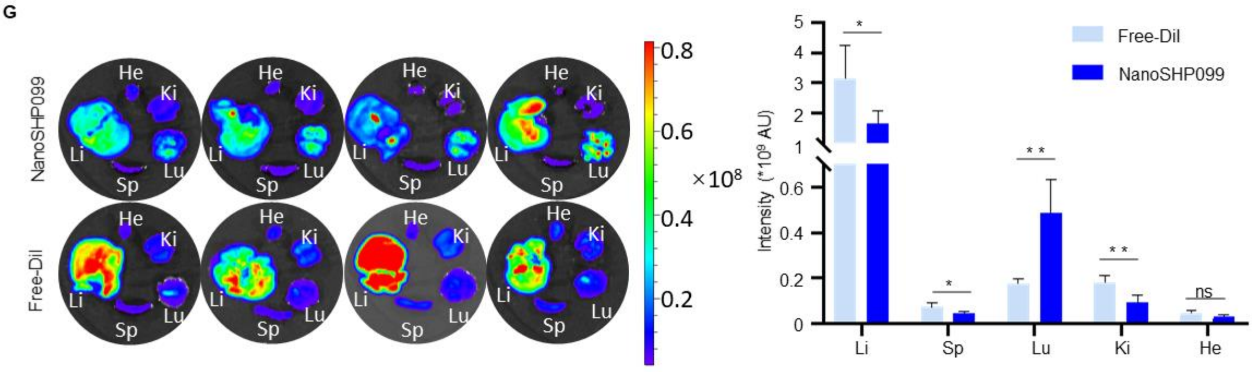

Supplementary Figure 2.

A. Cluster analysis heatmap and volcano plot showing NR4A and C/EBP genes family members differentially expressed in Ly6C^{high} and Ly6C^{low} monocytes/macrophages.

B. Volcanic Map of DEG in Ly6C^{high} and Ly6C^{low} monocytes/macrophages. Among the genes with log2 fold change >0 or <0, up- and down-regulated genes at $p < 0.05$ in the Ly6C^{low} monocytes/macrophages compared to Ly6C^{high} monocytes/macrophages were classified into DEGs and are represented by red and blue dots, respectively.

C. The display of the top 30 kinds proteins simultaneously interacted with NR4A1 and C/EBPβ.

D. Left: Contour plots of monocytes/macrophages (CD11B$^+$CD115$^+$) in freshly isolated bone marrow suspensions acquired from untreated SHP2$^{f/f}$, LysmCre-SHP2$^{f/f}$ mice.

Right: The statistics data of frequencies of bone marrow monocytes/macrophages analyzed by FCM. Data are shown as mean ± SD (n=3). *$P < 0.05$, **$P < 0.01$ and ***$P < 0.001$.

E. Left: Contour plots of monocytes/macrophages subsets, including CD11B$^+$CD115$^+$Ly6C^{high}, CD11B$^+$CD115$^+$Ly6C^{mediate}, CD11B$^+$CD115$^+$Ly6C^{high} in peripheral blood from untreated SHP2$^{f/f}$, LysmCre-SHP2$^{f/f}$ mice detected by FCM.

Right: Stacking histogram of proportions of peripheral blood Ly6C^{high}, Ly6C^{mediate} and Ly6C^{low} monocytes/macrophages subsets.

F. Left: Representative Masson and Sirius Red staining images of thrombus tissues obtained from SHP2$^{f/f}$, LysmCre-SHP2$^{f/f}$ DVT model mice at day 1 after inferior vena cava ligation. Data are shown as mean ± SD (n=4). *$P < 0.05$, **$P < 0.01$ and ***$P < 0.001$.

G. Left: *Ex vivo* fluorescence images of the DiI and NanoSHP099 in the major organs acquired from differently treated lung metastatic tumor model mice., Li, Sp, Lu, Ki and

He represent, liver, spleen, lung, kidney and heart, respectively.

Right: Quantitative analysis of the fluorescence intensity in each organ shown in the *ex vivo* images. Data is shown as mean ± SD (n=5). **P* < 0.05 and ***P* < 0.01.

FigS3.

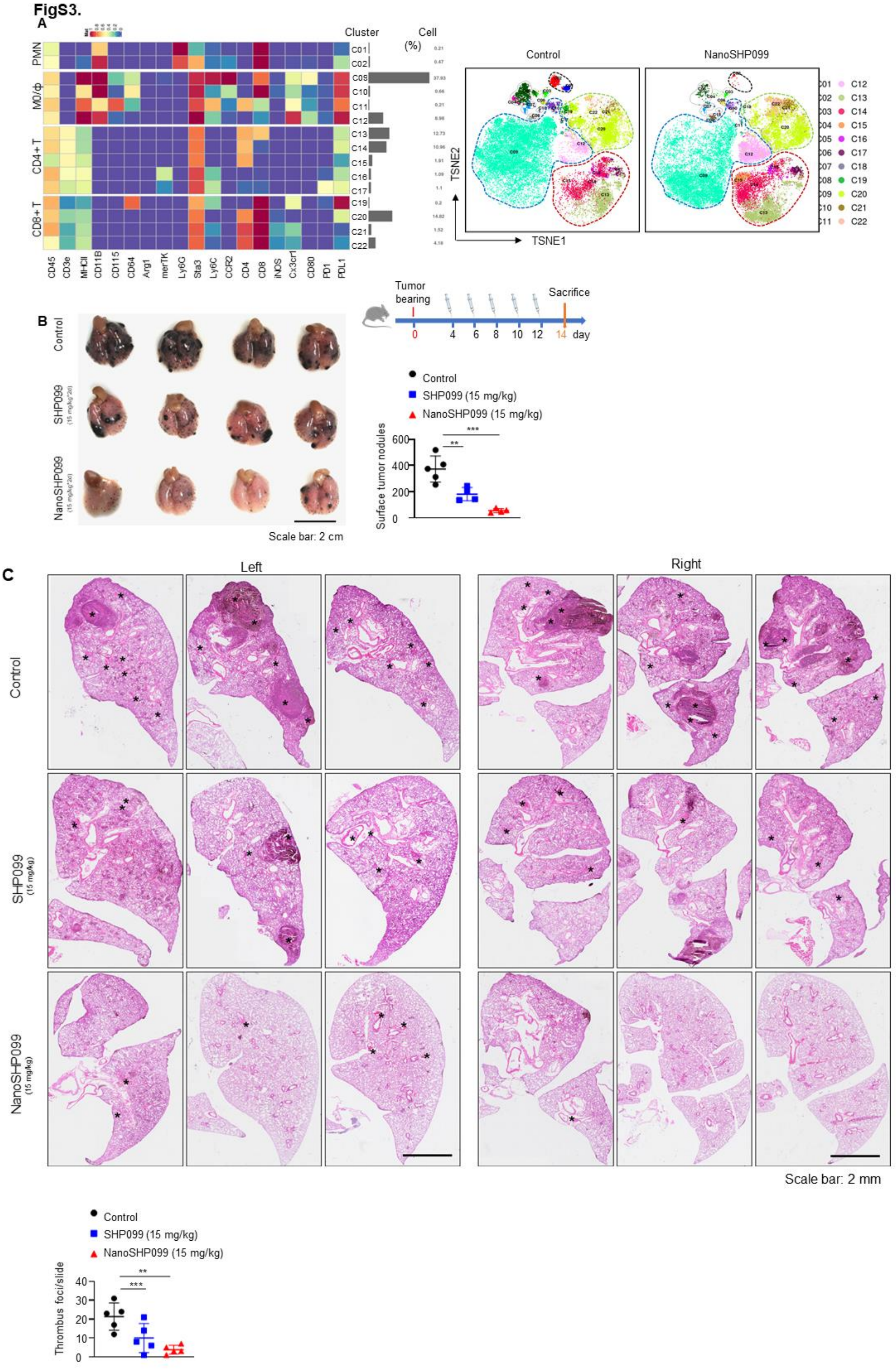
A
Cluster
Cell (%)
PMN
MΘ/Φ
CD4+ T
CD8+ T
C01
C02
C09
C10
C11
C12
C13
C14
C15
C16
C17
C19
C20
C21
C22
CD45
CD3e
MHCII
CD11B
CD115
CD64
Arg1
merTK
Ly6G
Sta3
Ly6C
CCR2
CD4
CD8
iNOS
Cx3cr1
CD80
PD1
PDL1
Control
NanoSHP099
TSNE1
TSNE2
B
Control
SHP099
NanoSHP099
Scale bar: 2 cm
Tumor bearing
Sacrifice
0
4
6
8
10
12
14
day
Control
SHP099 (15 mg/kg)
NanoSHP099 (15 mg/kg)
Surface tumor nodules
600
400
200
0
C
Left
Right
Control
SHP099 (15 mg/kg)
NanoSHP099 (15 mg/kg)
Scale bar: 2 mm
Thrombus foci/slide
40
30
20
10
0

Supplementary Figure 3.

A. Left: Heatmap showing the expression of marker genes in the indicated cell types.

Right: TSNE plots showing immune cell clusters in lung tissues infiltrated $CD45^+$ cells derived from lung metastases tumor model mice treated by vehicle or NanoSHP099. Neutrophils, monocytes/macrophages, $CD4^+$ T, and $CD8^+$ T were respectively circled by black, blue, red, and green dotted lines.

B. Left: Images of lungs acquired from lung metastatic tumor model mice after various drugs, including vehicle, SHP099 (15 mg/kg), NanOSHP099 (15 mg/kg) treatments every other day from day 4 after B16 injection for total 14 days.

Right: The quantitative analysis of metastatic tumor lesions on the surface of lung tissues. Data are shown as mean ± SD (n=5, 4). $*P < 0.05$, $**P < 0.01$ and $***P < 0.001$.

C. Above: HE staining images showing thrombus lesions (marked with *) in lung tissues acquired from lung metastatic tumor model mice after various treatments. Scale bar: 2 mm.

Below: The quantitative analysis of tumor-related thrombus foci located inside lung tissues. Data are shown as mean ± SD (n=5, 4). $*P < 0.05$, $**P < 0.01$ and $***P < 0.001$.

Supplementary Table 1.

Flow cytometric antibodies panels

Mouse Ly6C monocyte/macrophage subsets	Product No.
FITC anti-mouse/human CD11B	BioLegend Cat.101206
PE anti-mouse CD115 (CSF-1R)	BioLegend Cat.135505
APC anti-mouse Ly6C	BioLegend Cat.128015

Mouse Ly6C monocyte/macrophage subsets	Product No.
PE anti-mouse/human CD11B	BioLegend Cat.101207
PE/Cyanine7 anti-mouse CD115 (CSF-1R)	BioLegend Cat.135524
FITC anti-mouse CD192 (CCR2)	BioLegend Cat.150607
Alexa Flour 647 anti-mouse CX3CR1	BioLegend Cat.149004

Mouse monocyte/macrophages, neutrophils	Product No.
PE anti-mouse/human CD11B	BioLegend Cat.101207
FITC anti-mouse Ly6G	BioLegend Cat.127606
APC anti-mouse CD115 (CSF-1R)	BioLegend Cat.135509

Human monocyte subsets	Product No.
PE/Cyanine7 anti-mouse/human CD11B	BioLegend Cat.101215
PE anti-human CD115 (CSF-1R)	BioLegend Cat.347304
FITC anti-human CD14	BioLegend Cat.325603
APC anti-human CD16	BioLegend Cat.302011

Supplementary Table 2.

Antibodies applied in WB/ COIP/ IF/ IHC assays

Antibody	Species	Application	Product No.
Anti-CD115 (CSF-1R)	Rabbit	IHC/IF	CST Cat.3155S
Anti-Ly6G	Rabbit	IHC	CST Cat.87048S
Anti-SHP2	Rabbit	WB/COIP	CST Cat.3397S
Anti-SHP2	Goat	IF	Sigma Cat.SAB2500939
Anti-pSHP2	Rabbit	WB/IF	Abcam Cat.ab62322
Anti-CCR2	Rabbit	IF	Abcam Cat.ab273050
Anti-CX3CR1	Rabbit	IF	Abmart Cat.PY327135
Anti-C/EBPβ	Rabbit	COIP/WB	Abmart Cat.T552765
Anti-NR4A1	Rabbit	COIP/WB	Abmart Cat.T56890S
Anti-C/EBPβ (H-7)	Mouse	IF	Santa Cat.sc-7962
Anti-NR4A1 (c-5)	Mouse	IF	Santa Cat.sc-365113
β-actin	Mouse	WB	HuaAn Cat.M1210-2
IgG Isotype	Rabbit	COIP	Beyotime Cat.A7016
Anti-HA-Tag (26D11)	Mouse	COIP	Abmart Cat.M20003F
Anti-Flag-Tag	Mouse	COIP	Abmart Cat.M20008F
Anti-Myc-Tag (19C2)	Mouse	COIP	Abmart Cat.M20002F
680RD-anti-Mouse	Goat	WB	LICOR 926-68070
800CW-anti-Rabbit	Goat	WB	LICOR 926-32211
HRP-anti-Rabbit	Goat	IHC	HuaAn Cat.HA1001
Alexa Fluor 488 anti-Rabbit	Donkey	IF	Invitrogen Cat.A-21206
Alexa Fluor 555 anti-Mouse	Donkey	IF	Invitrogen Cat.A-31570
Alexa Fluor 647 anti-Goat	Donkey	IF	Invitrogen Cat.A-21447

Supplementary Table 3.

List of PCR primers, genotype identification, and siRNAs.

RT-qPCR primers：

Gene	Species	Sequences（5’-3’）
MMP2	Mouse	F-CAAGTTCCCCGGCGATGTC
		R-TTCTGGTCAAGGTCACCTGTC
MMP9	Mouse	F-CTGGACAGCCAGACACTAAAG
		R-CTCGCGGCAAGTCTTCAGAG
MMP12	Mouse	F-CTGCTCCCATGAATGACAGTG
		R-AGTTGCTTCTAGCCCAAAGAAC
MMP13	Mouse	F-CTTCTTCTTGTTGAGCTGGACTC
		R-CTGTGGAGGTCACTGTAGACT
MMP14	Mouse	F-CAGTATGGCTACCTACCTCCAG
		R-GCCTTGCCTGTCACTTGTAAA
MMP19	Mouse	F-CTGTGGCTGGCATTCTTACTT
		R-GGGCAGTCCAGATGCTTCC
MMP25	Mouse	F-TGGAATGGCTGACTCGCTATG
		R-GCATGACTTTGATTGCATCCTG
MMP28	Mouse	F-AACCAGAGGTCCTAAATACTGCC
		R-GGACGAGGCTCTACAGTGATG
TIMP2	Mouse	F-TCAGAGCCAAAGCAGTGAGC
		R-GCCGTGTAGATAAACTCGATGTC
TIMP3	Mouse	F-CTTCTGCAACTCCGACATCGT
		R-GGGGCATCTTACTGAAGCCTC
FGF	Mouse	F-GCGACCCACACGTCAAACTA
		R-CCGTCCATCTTCCTTCATAGC
EGF	Mouse	F-CGGGGAGTGCAGATACCTG
		R-TTCTCCACTGGTAGAGTCAGC

PDGF	Mouse	F-CATCCGCTCCTTTGATGATCTT
		R-GTGCTCGGGTCATGTTCAAGT
VEGF	Mouse	F-CTGCCGTCCGATTGAGACC
		R-CCCCTCCTTGTACCACTGTC
TGF	Mouse	F-CCACCTGCAAGACCATCGAC
		R-CTGGCGAGCCTTAGTTTGGAC
Actin	Mouse	F-AGAGGGAAATCGTGCGTGAC
		R-CAATAGTGATGACCTGGCCGT

Genotype identification：

Gene	Species	Sequences（5’-3’）
SHP2^{flox}	Mouse	F-ACGTCATGATCCGCTGTCAG
		R-ATGGGAGGGACAGTGCAGTG
Lysm-Cre	Mouse	F-CCCAGAAATGCCAGATTACG
		R-CTTGGGCTGCCAGAATTTCT

SiRNA：

Gene	Species	Sequences（5’-3’）
C/EBPβ	Human	F-CUGCCUUUAAAUCCAUGGATT
		R-UCCAUGGAUUUAAAGGCAGTT

Supplementary Table 4.

NR4A1 promoter sequence（C/EBPβbinding sites are highlighted in blue）

>NC_000012.12:52020832-52022832 Homo sapiens chromosome 12, GRCh38.p14 Primary Assembly
AGACAGAAAGGAAGCTGAGGGCTCTGGAGGGGCCACGGAAACCCAAGAGAGTTCCTCAAGAG
AGTAAAACAGCCAGAGTGCAGCAGTGTGCTGACCCCTGGTGGGGAGCCGGGACCATACCAGGT
GGAGAGGGGCACAGAGCAGGGCTCTCCCATCAGCCAACCATTGGGAGCAGCCTCCCTCCCTCC
TTAGAGCCCACCTCTTCAGCCCTTCCTCAGCCTGCTGCACGCTTGTACAAAGTGGAGCCTCCCCT
GAACCCCACAATCATTTATCTGAAGTGCTCAGACACCCTCCCACTGCTTCTGGTCCCAGGGCCTC
TCCTCACTCCACCACCCAGATTGTGGGCTCCCAAGGGAGTGAACCCCATCTCATGCCAGCACTG
ATCGAAGGCAGGTACGTCTGAATGAATGAATGAACAGTTGAGTTCAGAACATCTAACTAAATGA
CTAAATGACTCCAACCAACAGACCTTCTCTGCACAGAACCCTGTGTGGTATGAAAGCTCATGGA
CTGGGGTTGATAGTGGAGAGGAAACATACTTTTCAACAGCAAGATGCAAGAGTGTTTTTATTATT
TATTTATAATTTATTTTTATTTTTAGAGATGTGTCTTGCATGCAATCAATATGGGGACCTCCCAGGA
GCAGGAGGCCACCAG**GTTGCCTAAGA**CATATGAATTTTTGCTTTCAGGGACACCTCCTAAGCTT
CAGTGATCCATCTTGGAGTTGGCCTTTGTGGGGAGAACATATAAATTTATGCAAAAAGTAAAAAT
GCAAATACTGCCTCTGGGTCTCTTTCCCAGCTGCTCCCCTACAGAGTCACTTAGCCTTTAGTGAC
TCACAGTTGATCTGGTCCACTCAGCAACCCACAGAGTGACAGCCCACCCAGGTCCCAGCATACA
TAGCATCTTCTTCTGGAGCCCCCCAAATCTCACTCTAATTGAGGGCTTGGCTGGAGCCACTCTCT
CTGAGGAGTGTGGTCTCTGGGCAAATAAGGCAAGACCAGTGGCTGGCAGCTACAGCAGTCAGT
AGGGTGGGGGACATTTAACAGCAGATAGGGCCATACTCCTACTCTTGCCATTCTTTTTACCCTTCC
CCTCCCCCTTGCAATTCTTTTCTGTTTTTTTTTTTTTTTTTGGTTTTTGAAACAGGGTCAGCTGGG
TGCGGTGGCTCACGCCTGTAATCCCAGCACTTTGGGAGGTTGGTAGATCACTTGAGGTCAGGAG
TTCAAGACCAGACTGGACAACACAGTGAAACCCAGTCTCTACTAAAAATATAAAAATTAGCTGG
GCGTGGTGGCACATGCCTGTAAACCCAGCTACTGGGGATGCTGAGACAGGAGAATCGCTTGAA
GCTGGGAGGTGGAGGTTGCAGCGAGCAGAGATTGGGACACTGCACTCCAGCCTGGGCAACAG
AGCGAGACTCTGTCTCAAAAAAAGAAAAAGAAAAAGAAAAAGAAAGAAAGAAAGAAAAAAA
AAGAAACAGGAAACAGGGTCTTGCTCTGTCGTCCAACCTGGATGCAGTGGTATAATCACAGCTC
ACCGCAGCCTCAATCTCCTGGGTTTAAGCGATCCTCAACGTTTGGAGTAGCTGGGACTGCAGGC
GTAGGCCACCACCCCAGGCTAATTTTTGTATTTTTTGTAGAGAGA**GGTTTCGCCATG**TTGGCCA
GGCTGGTCTCAAACTCCTGGGCTCAAGCAATTCTCCTAACTCGGCCTTCCAAAGTGCTGGGATT
ACAGGTGTGAGCCACCACTCCTGGCCCCCTTGAGATTCTGATCCAGGAGTTCTAGGGTGGGCTG
GGGCGTTGGTATTTTTAAAGCACCCTAGGTGCTTAGTGAGTCTTTATTGAAAGGATGTTGACTGA
AGATAATTCTGGAGATCTCTGAGCTCCCTCCCACACAGATCCTTGGGACTCTAAATACTGGCCGA
ACTCGGCCCCCTGAGGCTGTGTCTTCTTGGGGAGCTAAGGACCTGTGCCTCCCACTGGCCCGGA
ACCACTTTCTGCT
